# miR-458b-5p regulates ovarian granulosa cells proliferation through Wnt/β-catenin signaling pathway by targeting catenin beta-1

**DOI:** 10.5713/ajas.20.0392

**Published:** 2020-10-13

**Authors:** Wenwen Wang, Jun Teng, Xu Han, Shen Zhang, Qin Zhang, Hui Tang

**Affiliations:** 1 Shandong Provincial Key Laboratory of Animal Biotechnology and Disease Control and Prevention, Shandong Agricultural University, Taian, Shandong 271018, China

**Keywords:** miR-458b-5p, Catenin Beta-1 (*CTNNB1*), Granulosa Cells, Proliferation, Wnt, β-catenin Signaling Pathway

## Abstract

**Objective:**

Ovarian follicular development, which dependent on the proliferation and differentiation of granulosa cells (GCs), is a complex biological process in which miRNA plays an important role. Our previous study showed that miR-458b-5p is associated with ovarian follicular development in chicken. The detailed function and molecular mechanism of miR-458b-5p in GCs is unclear.

**Methods:**

The luciferase reporter assay was used to verify the targeting relationship between miR-458b-5p and catenin beta-1 (*CTNNB1*), which is an important transcriptional regulatory factor of the Wnt/β-catenin pathway. The cell counting kit-8 (CCK-8) assay, flow cytometry with propidium iodide (PI) and annexin V-fluorescein isothiocyanate (FITC) labeling were applied to explore the effect of miR-458b-5p on proliferation, cell cycle and apoptosis of chicken GCs. Quantitative real-time polymerase chain reaction and Western blot were used to detect the mRNA and protein expression levels.

**Results:**

We demonstrated that the expression of miR-458b-5p and *CTNNB1* showed the opposite relationship in GCs and theca cells of hierarchical follicles. The luciferase reporter assay confirmed that *CTNNB1* is the direct target of miR-458b-5p. Using CCK-8 assay and flow cytometry with PI and Annexin V-FITC labeling, we observed that transfection with the miR-458b-5p mimics significantly reduced proliferation and has no effects on apoptosis of chicken GCs. In addition, miR-458b-5p decreased the mRNA and protein expression of CD44 molecule and matrix metallopeptidase 7, which are the downstream effectors of *CTNNB1* in Wnt/β-Catenin pathway and play functional roles in cell proliferation.

**Conclusion:**

Taken together, the data indicate that miR-458b-5p regulates ovarian GCs proliferation through Wnt/β-catenin signaling pathway by targeting *CTNNB1*, suggesting that miR-458b-5p and its target gene *CTNNB1* may potentially play a role in chicken ovarian follicular development.

## INTRODUCTION

Growth of the ovarian follicle in preparation for ovulation requires coordinated expression by granulosa cells (GCs) in response to autocrine, paracrine and endocrine factors [1]. The dynamic and highly regulated process requires the coordinated actions of a great number of genes, which is orderly orchestrated at the transcriptional and post-transcriptional levels [2]. While some of genes like Wnt family member 4 [3], parathyroid hormone like hormone [4], cytochrome P450 family 11 subfamily A member 1 [5] are known to be related to follicle development, new regulators continue to be uncovered. With the discovery of small RNAs that exert additional layers of control on gene expression, studies have begun looking at the roles of these molecules in regulating cellular events in the ovary.

MicroRNAs (miRNAs) are a class of endogenous small, non-coding RNAs. They are expressed in a time- and tissue-specific manner in even the most primitive animals, and show a great deal of conservation among a diverse range of species [6]. miRNAs play an integral role in many different biological processes including cell proliferation, differentiation, development, apoptosis, tumorigenesis, lipogenesis and host response [7–11]. Increasing evidence supports the vital role of miRNAs in the animal gonad by guarding genomes and guiding development [12,13], and miRNAs are crucial for controlling cell proliferation, differentiation, steroidogenesis, and apoptosis in the ovary [14]. Many miRNAs are expressed in GCs and directly regulate normal development and function of ovarian follicles [15], including the formation of primordial follicles, follicular recruitment and selection, follicular atresia, oocyte-cumulus cell interaction, GC function and luteinization by targeting specific molecules and modulating various signaling pathways, such as TGFB-, FSH-, hormone- and apoptosis-related pathways [16,17]. Single nucleotide polymorphisms (SNPs) in miRNAs, including pri-miRNA (primary miRNA transcripts), pre-miRNA (precursor miRNA) and mature miRNA are associated with the abundance of mature miRNA and may contribute to phenotypic variation in animals [13].

Ovarian follicular development is dependent on the pro liferation and differentiation of GCs [18]. Several factors regulating the proliferation and differentiation of GCs have been reported, most of which belong to the transforming growth factor superfamily, for instance the group of bone morphogenetic proteins (BMPs) [19]. Members of the Wnt family are secreted glycoproteins that were recently identified as regulators of ovarian function [3]. Wnt proteins may act through β-catenin-dependent or β-catenin-independent pathways and their abnormal expression and activation may cause tumors [20]. The β-catenin-dependent pathway is involved in the regulation of cell proliferation, cell fate determination, and embryonic induction [21]. Wnt signaling pathways are critical for ovarian development and essential for normal follicle development [22]. β-Catenin, encoded by catenin beta-1 (*CTNNB1*) gene, is the key mediator of canonical Wnt/β-catenin signaling, which activates the transcription of vital target genes responsible for cell proliferation [23]. Therefore, the regulated expression of β-catenin plays an important role in fertility. Our previous mRNA and miRNA transcriptome study found that *CTNNB1* acts as a candidate target of miR-458b-5p and potentially associated with ovarian follicular development in chicken [24].

Our current study sought to investigate potential regula tion exerted by miR-458b-5p on *CTNNB1* expression using chicken ovarian GCs as a model.

## MATERIALS AND METHODS

### Animals

Sexually mature (300 days old) Hyline-brown hens were randomly selected from the local research farm affiliated with Shandong Agricultural University. All chickens had free access to water and feed. Hens were sacrificed by decapitation. All of the animal experiments were approved by the Institutional Animal Care and Use Ethics Committee of Shandong Agricultural University (SDAUA-2018-018) and performed in accordance with the “Guidelines for Experimental Animals” of the Ministry of Science and Technology of China.

### Cell culture

The GCs and theca cells (TCs) isolation were conducted according to previous protocol [13,25]. Briefly, the hen ovaries were isolated and placed in phosphate buffered saline (HyClone, Logan, UT, USA). The granulosa layer was separated from the hierarchical follicle and then gently agitated in a flask with 0.2% (w/v) collagenase II (Gibco, Grand Island, NY, USA) at 37°C for 10 min. The theca layer was dissected from surrounding tissues and placed in 0.2% (w/v) collagenase II at 37°C for 30 min with gentle agitation in a flask. After centrifugation, the cells were suspended in culture medium (M199 with 10% fetal bovine serum and 1% penicillin/streptomycin), and subsequently seeded in 24-well culture plates at a density of 2×10^5^/well. The number of cells was estimated using Trypan blue. Cells were cultured at 38°C in a water-saturated atmosphere of 5% CO_2_.

### miRNA target gene prediction and luciferase reporter assay

The miRNA target genes were predicted using TargetScan 7.2 (http://www.targetscan.org/) and miRDB (http://mirdb.org/). The free energy of the miR-458b-5p-*CTNNB1* interaction was calculated using RNAhybrid 2.2 [26]. The sequence of *CTNNB1* 3′ untranslated regions (UTR) containing the putative miR-458b-5p binding region was amplified and cloned into pmirGLO dual-luciferase miRNA target expression vector. The potential miR-458b-5p binding sites were mutated by the Fast Site-Directed Mutagenesis Kit (TIANGEN, Beijing, China). GCs were cotransfected with the wild-type or mutant 3′UTR luciferase reporter plasmids, and the miR-458b-5p mimics or negative control (NC), respectively. The GCs were grown in 24-well plates to 80% confluence and transfected using Lipofectamine 2000 (Invitrogen, Shanghai, China) according to the manufacturer’s instruction. Cells were harvested 24 h after transfection, and the luciferase activities were measured using the Dual-Glo Luciferase Assay System (Promega, Madison, WI, USA). Firefly luciferase was normalized to Renilla activity.

### Cell proliferation

Cell proliferation was measured with the Cell Counting Kit-8 (Dojindo, Tokyo, Honshu, Japan), according to the manufacturer’s instructions. Cells were seeded in 96-well plates, the absorbance at 450 nm was measured after transfection 0, 12, 24, 36, 48, and 60 h with Infinite M200 Pro (Tecan, Männedorf, Switzerland) after a 2h incubation.

### Cell cycle analysis

The chicken GCs were cultured in six-well plates in triplicate and treated by miR-458b-5p mimics or mimics NC for 48 h. The cells were harvested and washed in phosphate-buffered saline (PBS) and fixed in ice-cold ethanol overnight at 4°C. The fixed cells were washed in PBS and stained with 50 μg/mL propidium iodide (PI) contain 50 μg/mL RNase A (DNase free) for 20 min at room temperature. Then the stained cells were examined by fluorescence-activated cell sorting (BD Biosciences, San Diego, CA, USA). The percentage of the cells in G1, S, and G2 phase were analyzed using FlowJo v7.6 software (Stanford University, Stanford, CA, USA).

### Cell apoptosis analysis

The GCs were seeded into six-well plates in triplicate and transfected with miR-458b-5p mimics or mimics NC for 2 days. The apoptosis rate was detected by flow cytometry (BD Biosciences, USA) using the Annexin V-fluorescein isothiocyanate (FITC) apoptosis detection kit (Beyotime, Shanghai, China), according to the manufacturer’s instructions. Quantification of apoptosis was determined by FlowJo v7.6 software (Stanford University, USA). The apoptosis rate was calculated using the following equation: (number of cells in the right lower quadrant + number of cells in the right upper quadrant)/(total number of cells).

### RNA isolation and quantitative real-time polymerase chain reaction

For coding gene detection, total RNA was extracted from cells using TRIzol kit (Invitrogen, Chinal) and digested with RNase-free DNase I according to the method described before [27]. Total RNA was reverse transcribed into complementary DNA (cDNA) using PrimeScript RT reagent Kit (TaKaRa, Dalian, China). For miRNA detection, miRNA was harvested using miRcute miRNA isolation kit (TIANGEN, China) and was reverse-transcribed into cDNA by using Mir-X miRNA First-Strand Synthesis Kit (TaKaRa, China). Quantitative real-time polymerase chain reaction (qRT-PCR) was performed in triplicate using TB Green Premix Ex Taq (TaKaRa, China) on a LightCycler 480 (Roche, Basel, Switzerland). The 2^−ΔΔCt^ method was used to calculate the relative gene and miRNA expression normalized by glyceraldehyde-3-phosphate dehydrogenase and U6 small nuclear RNA (U6), respectively. U6 forward primer, U6 reverse primer and common miRNA downstream primer (mRQ 3′ primer) are supplied with the Mir-X miRNA First-Strand Synthesis Kit. The other primer sequences used for qRT-PCR are listed in Table 1.

### Western blot

Cells were lysed with RIPA lysis buffer (PPLYGEN, Beijing, China) and protein concentrations were quantified using bicinchoninic acid protein assay kit II (BIO-RAD, Hercules, CA, USA). The total cellular protein was separated by 10% sodium dodecyl sulfate-polyacrylamide gel electrophoresis gel and then transferred to a polyvinylidene fluoride membrane (Millipore, Billerica, MA, USA). The membrane was blocked with 5% skim milk for 3 h and incubated with primary antibody overnight at 4°C. After that, the membrane was washed thrice for 10 min each time using tris-buffered saline with tween-20 and incubated with the appropriate secondary antibodies. The primary antibodies used for Western blot include rabbit anti-β-catenin (ab6302, Abcam, Cambridge, UK), mouse anti-CD44 (8400-08, SouthernBiotech, Birmingham, AL, USA) and rabbit anti-MMP7 (K006648P, solarbio, Beijing, China). Mouse anti-β-actin (sc-47778, Santa Cruz Biotechnology, Dallas, TX, USA) was used as a loading control. Secondary antibodies include goat anti-rabbit immunoglobulin G (IgG, A0208, Beyotime, China) and goat anti-mouse IgG (A0216, Beyotime, China). The blot signal was visualized using ECL reagent (Thermo Fisher Scientific, Waltham, MA, USA). The protein expression level was quantified by the band density using Quantity One 4.6.3 software (Bio-Bad, Hercules, CA, USA) and normalized by β-actin.

### Statistical analysis

All results are presented as the mean±standard deviation of at least three independent experiments. Statistical analyses were performed using GraphPad Prism software (version 7.0; San Diego, CA, USA). Comparison between two groups was analyzed using a Student-t test. ANOVA method was used to compare the data from more than two groups. Statistical significance is defined when p values are less than 0.05.

## RESULTS

### Expression characteristics of miR-458b-5p and *CTNNB1* in follicles

**Figure 1:**
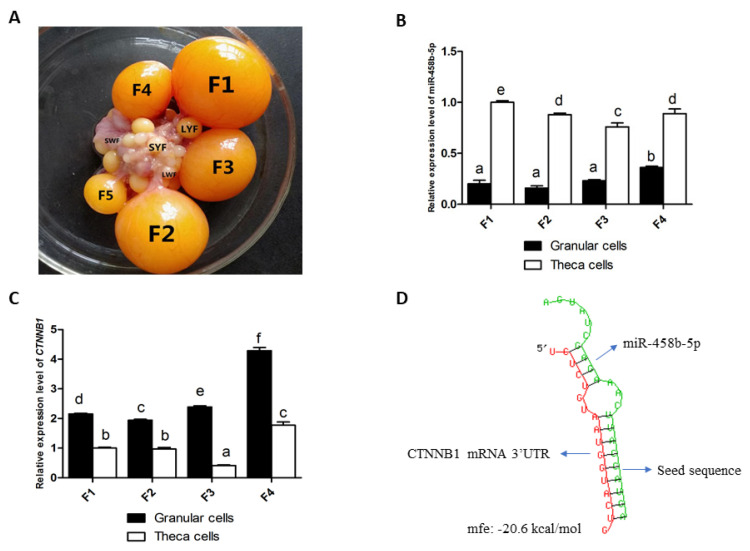
Expression patterns of miR-458b-5p and *CTNNB1* during follicular development. (A) Hierarchical follicles were separated from Hyline brown layer. (B) Expression levels of miR-458b-5p in the GCs and TCs of hierarchical follicles. (C) Expression levels of *CTNNB1* in the GCs and TCs of hierarchical follicles. (D) Binding site prediction between the miR-458b-5p sequence and *CTNNB1* 3′UTR using RNAhybrid 2.2. *CTNNB1*, catenin beta-1; GCs, granulosa cells; TCs, theca cells; UTR, untranslated region. Data are presented as the mean±standard error of the mean from at least three independent experiments. Bars with different letters are significantly different (p<0.05).

To study the role of miR-458b-5p in ovarian follicular development, we examined the expression of miR-458b-5p in GCs and TCs of hierarchical follicles using qRT-PCR. The F1–F4 hierarchical follicles were selected from Hyline brown layers (Figure 1A). miR-458b-5p was expressed in both GCs and TCs of all the hierarchical follicles, but the expression levels in the TCs were significantly higher than that in GCs (p<0.05) (Figure 1B). In the TCs, the miR-458b-5p expression level was found to be highest in the F1 follicles, but in the GCs, the miR-458b-5p was highest in the F4 follicles. Then, we identified the mRNA expression pattern of *CTNNB1* during follicles development by qRT-PCR. In contrast, the mRNA expression of *CTNNB1* in the TCs were significantly lower than that in the GCs (p<0.05) (Figure 1C). F4 follicles have the highest mRNA expression levels of *CTNNB1* both in TCs and GCs. To explore the possible miRNA:mRNA regulatory mechanism, the TargetScan and miRDB algorithms were used to predict targeting regulatory relationship of miR-458b-5p and *CTNNB1*. A total of 623 and 314 targets of miR-458b-5p were predicted with TargetScan and miRDB, respectively (Supplementary Table S1, S2), and 139 genes overlapped with the targets (Supplementary Table S3). Also, 113 and 39 miRNAs were predicted targeting *CTNNB1* using TargetScan and miRDB, respectively (Supplementary Table S4, S5), and 33 miRNAs overlapped with the miRNAs (Supplementary Table S6). *CTNNB1* was predicted as a potential target gene of miR-458b-5p both by TargetScan and miRDB (Supplementary Table S3, S6), with an estimated free energy of −20.6 kcal/mol for the interaction between them (Figure 1D). With a potentially conserved miRNA:mRNA regulatory mechanism, we sought to investigate potential regulation exerted by miR-458b-5p on *CTNNB1* using chicken ovarian GCs as model.

### miR-458b-5p down regulated *CTNNB1* gene expression via targeting its 3′UTR

**Figure 2:**
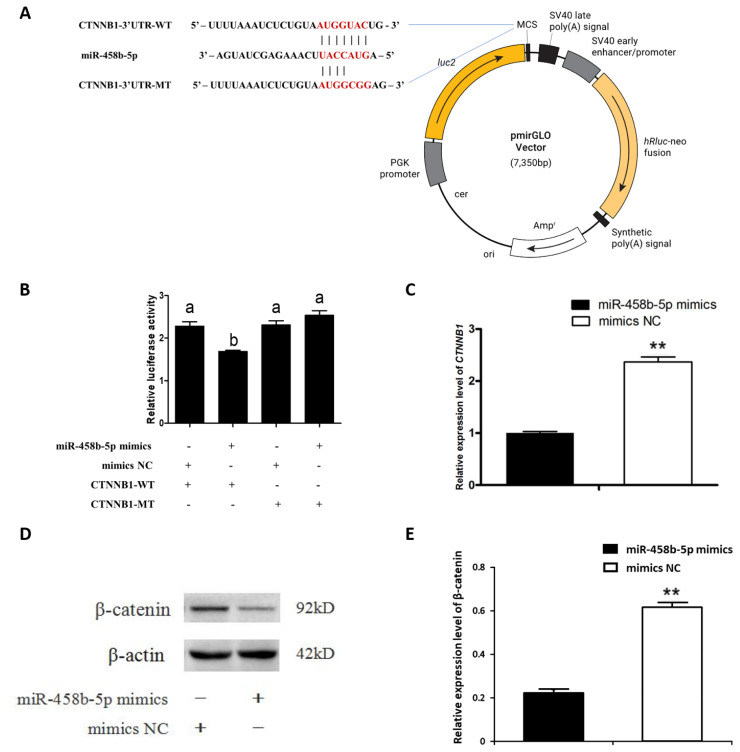
miR-458b-5p directly regulates *CTNNB1*. (A) Schematic diagram of the dual luciferase reporter pmir-GLO-CTNNB1-3′UTR. (B) Dual luciferase assays were performed by co-transfection of miR-458b-5p mimics or mimics NC and wild-type vectors or mutant vectors. (C) The relative *CTNNB1* mRNA expression levels after treatment with the miR-458b-5p mimics. (D) Western blot analysis of β-catenin protein expression after treatment with the miR-458b-5p mimics. (E) The quantification of β-catenin protein levels. *CTNNB1*, catenin beta-1; WT, wild-type; MT, mutant-type; UTR, untranslated regions; MCS, multiple cloning site; NC, negative control. Results are presented as means±standard error of the mean of three independent determination, ** p<0.01. Bars with different letters are significant different (p<0.05).

We investigated the relationship between miR-458b-5p and the *CTNNB1* 3′UTR using a luciferase reporter system. *CTNNB1* 3′UTR wild-type (WT) and putative interaction region mutant-type (MT) vector were constructed (Figure 2A) and co-transfected with miR-458b-5p mimics into chicken ovarian GCs. A dual luciferase reporter assay showed that miR-458b-5p mimics significantly decreased luciferase activity of the *CTNNB1* 3′UTR-WT but did not affect the luciferase activity of the *CTNNB1* 3′UTR-MT (Figure 2B). Then we examined the mRNA (Figure 2C) and protein levels (Figure 2D, 2E) of *CTNNB1* and found a remarkable decrease in the treatment of chicken ovarian GCs with miR-458b-5p mimics. Taken together, these data showed that miR-458b-5p was directly targeting *CTNNB1* and inhibiting the expression of *CTNNB1*.

### miR-458b-5p inhibits the proliferation of chicken ovarian granulosa cells

**Figure 3:**
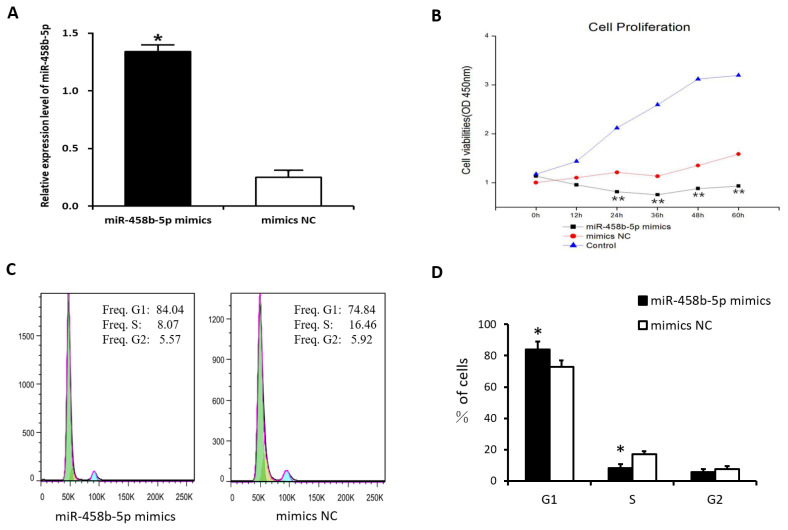
miR-458b-5p inhibits the proliferation of chicken ovarian GCs. (A) Transfection with the miR-458b-5p mimics remarkably increased the miR-458b-5p expression level after transfection 48h. (B) Chicken ovarian GCs proliferation estimated by CCK8 assay in response to overexpression of miR-458b-5p after transfection 0, 12, 24, 36, 48, and 60 h. (C) Cell cycle was detected in chicken GCs 2 days after transfection. (D) Histogram represented the percentage of cells in the G1, S, and G2 phases. GCs, granulosa cells; CCK-8, cell counting kit-8. * p<0.05.

To determine the role of miR-458b-5p on cell proliferation, chicken ovarian GCs were transfected with miR-458b-5p mimics, mimics NC, and blank control. The result showed that the transfection with the miR-458b-5p mimics remarkably increased the miR-458b-5p expression level after transfection 48 h (Figure 3A). Then, the cell proliferation in the chicken ovarian GCs was estimated using CCK8 assay after transfection 0, 12, 24, 36, 48, and 60 h. The results showed that the overexpression of miR-458b-5p significantly decreased the viability of GCs in a time-dependent manner compared to the mimics NC (Figure 3B). Because the cell cycles are involved in the regulation of cell proliferation, we analyzed the processed cells with a flow cytometer. Our results revealed that cell cycles were arrested significantly at the G1 stage in the miR-458b-5p mimics group (Figure 3C, 3D). These results suggest that miR-458b-5p may suppress cell proliferation of chicken GCs.

### miR-458b-5p have no effects on apoptosis of chicken ovarian granulosa cells

**Figure 4:**
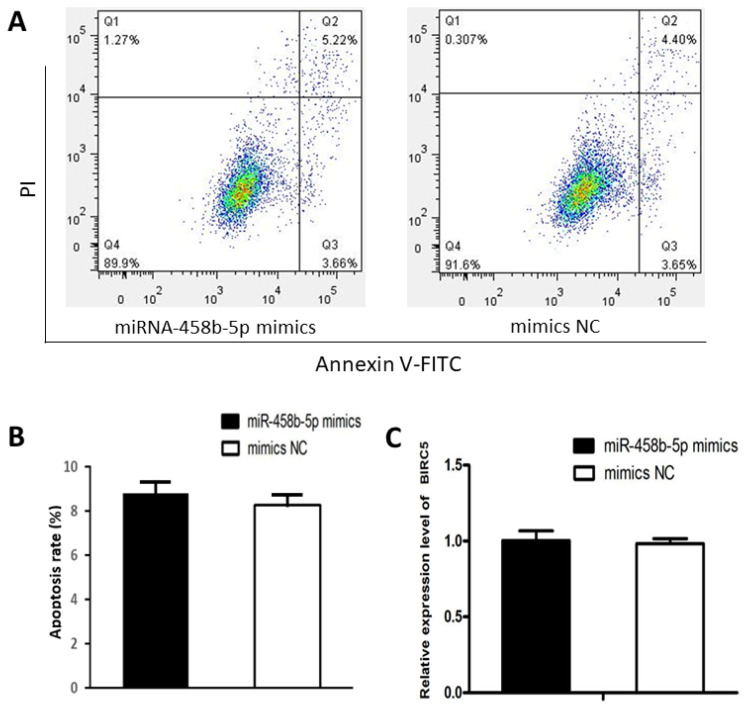
miR-458b-5p does not have specific effects on apoptosis of chicken ovarian GCs. (A) The GCs apoptosis rate was detected by fluorescence-activated cell sorting (FACS). (B) The apoptosis was calculated. (C) The mRNA expression levels of *BIRC5* in chicken ovarian GCs after transfected with miR-458b-5p mimics or mimics NC. GCs, granulosa cells; *BIRC5*, baculoviral IAP repeat containing 5; NC, negative control. Each experiment has three independent repetition and results are shown as mean±standard error of the mean.

We examined the apoptosis rate in chicken ovarian GCs transfected with miR-458b-5p mimics compared with mimics NC. Flow cytometry with the Annexin V-FITC labeling revealed that there was no significant difference between the two groups in respect to cell apoptosis (Figure 4A, 4B). Furthermore, the mRNA expression levels of baculoviral IAP repeat containing 5 (*BIRC5*), an evolutionarily conserved eukaryotic protein that can inhibit cell apoptosis [28], showed no significant difference between the two groups (Figure 4C), indicating that miR-458b-5p does not have specific effects on the cell apoptosis of chicken ovarian GCs.

### miR-458b-5p suppress the β-catenin signaling pathway

**Figure 5:**
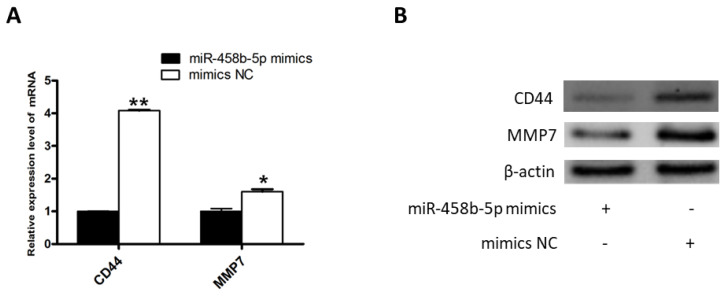
miR-458b-5p suppresses the expression of downstream genes of β-catenin signaling. (A) The mRNA expression levels of *CD44* and *MMP7* were measured by qRT-PCR. (B) The expressions of CD44 and MMP7 proteins were measured by Western blot. *CD44*, CD44 molecule; *MMP7*, matrix metallopeptidase 7; qRT-PCR, quantitative real-time polymerase chain reaction. Data are means of at least three independent experiments; ** p<0.01, * p<0.05.

To further investigate the possible molecular mechanisms of miR-458b-5p induced proliferation repression, we examined the mRNA and protein expression levels of downstream pathway regulators of β-catenin after transfection with the miR-458b-5p mimics and mimics NC. The results showed that mRNA and protein expression of CD44 molecule (*CD44*) and matrix metallopeptidase 7 (*MMP7*), two well-known targets of β-catenin [29,30], were appreciably down-regulated when miR-458b-5p increased (Figure 5A, 5B). It is reported that *CD44* is a transmembrane receptor responsible for cell proliferation [31]. *MMP7* promote cell proliferation by degrading E-cadherin and thereby liberating β-catenin [30]. Collectively, these data showed that miR-458b-5p may suppress ovarian GCs proliferation by regulating β-catenin signaling pathway.

## DISCUSSION

Ovarian follicular development is important for egg production and laying performance. Follicular development is dependent on the proliferation and differentiation of GCs [18]. Understanding the molecular regulatory patterns of GCs is crucial to understand the laying mechanism and improving the laying performance of hens. Several factors, such as BMPs [19] and Wnts [3], regulating the proliferation and differentiation of GCs have been studied. Besides, microRNAs were previously shown to regulated GCs proliferation [32].

Our previous miRNA transcriptome study found that miR-458b-5p is associated with ovarian follicular development in chicken [24]. However, no research has been conducted on miR-458b-5p function in regulating GCs proliferation. In this study, we demonstrated that miR-458b-5p inhibits proliferation and has no effect on apoptosis of chicken GCs. Using dual luciferase reporter assay, we also identified and validated that *CTNNB1* gene was a direct target of miR-458b-5p.

β-Catenin, encoded by *CTNNB1* gene, is a crucial regulator in the Wnt/β-catenin signaling pathway. Numerous studies have demonstrated that Wnt/β-catenin signaling pathway plays an important role in the regulation of cell proliferation, invasion, metastasis, apoptosis, differentiation, and so on. Our results demonstrated that miR-458b-5p decreased *CTNNB1* mRNA and protein expression (Figure 2C–2E), inhibits proliferation (Figure 3) and has no effect on apoptosis (Figure 4) of chicken GCs. The results were similar with previous study, which demonstrated that knockout of *CTNNB1* suppressed proliferation and does not have an effect on apoptosis of 293T cells [23]. Moreover, the downstream genes of Wnt/β-catenin signaling pathway were reported to be overexpressed when the Wnt/β-catenin signaling is activated in various cell lines [33]. Then we detected the β-catenin target genes, *CD44* and *MMP7*, of chicken GCs transfected with miR-458b-5p mimics and mimics NC. As we expected, the mRNA and protein expression levels of *CD44* and MMP7 declined when β-catenin was down-regulated by miR-458b-5p. It is reported that *CD44* plays a functional role in *Helicobacter pylori*-induced gastric epithelial cell proliferation both *in vitro* and *in vivo* [31]. In addition, *MMP7* promotes cell proliferation of lung adenocarcinoma cells and colon cancer cells [30]. Based on these, we propose that miR-458b-5p regulates chicken GCs proliferation via Wnt/β-catenin signaling pathway by targeting *CTNNB1*. The involvement of miR-458b-5p and *CTNNB1* in regulation of chicken GCs proliferation makes them potential candidates as biomarkers or targets for egg production. Further characterization of SNPs in miR-458b-5p and *CTNNB1* will clarify the potential contribution of polymorphisms to ovarian function in chickens. Also, with technological progress, targeted knockdown or delivery of miRNA or genes to ovaries may provide a potential approach for the improvement of egg production and laying performance in the poultry industry.

In conclusion, our study proved the mechanism of miR- 458b-5p suppresses chicken GCs proliferation by inhibiting β-catenin at the post-transcriptional level and blocking the Wnt/β-catenin signaling pathway with the result that the downstream cell proliferation related genes could not be activated. Therefore, miR-458b-5p and its target gene *CTNNB1* may potentially play a role in chicken ovarian follicular development.

## Figures and Tables

**Table 1:** Primer sequences

Genes	Primers	Annealing (°C)
*CTNNB1*	Forward primer: 5′-CCGAAACACTGGATGAAGGA-3′ Reverse primer: 5′-GCTGATGAACCATAACCGCA-3′	54
*BIRC5*	Forward primer: 5′-GAATGGCTGGTCTACCTCGT-3′ Reverse primer: 5′-CACCGTCAGGTTAGAGGGAT-3′	56
*CD44*	Forward primer: 5′-ATCAGGGACCACACAAGGGA-3′ Reverse primer: 5′-ACTTGCTGGCATCTCCGTTT-3′	60
*MMP7*	Forward primer: 5′-GTTACCTCGGGACAGGCAGA-3′ Reverse primer: 5′-CAGGGCTCCACGGACATTTG-3′	60
*GAPDH*	Forward primer: 5′-GCTGATGCTCCCATGTTCGTGAT-3′ Reverse primer: 5′-GTGGTGCAAGAGGCATTGCTGAC-3′	61
miR-458b-5p	Forward primer: 5′-AGTACCATTCAAAGAGCTATGA-3′	60

*CTNNB1*, catenin beta-1; *BIRC5*, baculoviral IAP repeat containing 5; *CD44*, CD44 molecule; *MMP7*, matrix metallopeptidase 7; *GAPDH*, glyceraldehyde-3-phosphate dehydrogenase.
