# Supplementary material for: miR-458b-5p regulates ovarian granulosa cells proliferation through Wnt/β-catenin signaling pathway by targeting catenin beta-1
Source: Anim Biosci. 2020 Oct 13;34(6):957–66. doi: 10.5713/ajas.20.0392 (PMC8100484; doi:10.5713/ajas.20.0392)
Supplement: Supplementary file 1 [file ajas-20-0392-suppl.pdf]

Table S1 miR-458b-5p target genes predicted using TargetScan

Target gene	Representative transcript	Gene name	3P-seq tag	Total sites	8mer sites	7mer-m8 site
PLA2G15	ENST00000566188.1	phospholipas	2670	3	1	2
DPYSL3	ENST00000398514.3	dihydropyrin	5209	1	1	0
SOWAHC	ENST00000356454.3	sosondowah	431	1	1	0
CARF	ENST00000402905.3	calcium resp	27	2	1	1
POFUT2	ENST00000349485.5	protein O-fuc	842	1	1	0
YES1	ENST00000577961.1	v-yes-1 Yam	168	2	2	0
PTN	ENST00000393083.2	pleiotrophin	11	1	1	0
CASP2	ENST00000310447.5	caspase 2, ap	274	1	1	0
BCL7A	ENST00000538010.1	B-cell CLL/l	96	1	1	0
KIAA1715	ENST00000272748.4	KIAA1715	858	1	1	0
ARL8B	ENST00000419534.2	ADP-ribosyl	664	2	2	0
PHIP	ENST00000275034.4	pleckstrin ho	668	2	2	0
CLIC4	ENST00000374379.4	chloride intr	226	1	1	0
KREMEN1	ENST00000400335.4	kringle conta	606	1	1	0
ANKRD44	ENST00000282272.8	ankyrin repe	187	1	1	0
CXCR5	ENST00000292174.4	chemokine ((	5	1	1	0
GAB2	ENST00000340149.2	GRB2-associ	627	1	1	0
AC019294.1	ENST00000543887.1		5	1	1	0
CDC20B	ENST00000334206.5	cell division	5	1	1	0
SLC35E1	ENST00000595753.1	solute carrier	4273	1	1	0
MPST	ENST00000404393.1	mercaptopyru	145	1	1	0
LIN28B	ENST00000345080.4	lin-28 homol	118	2	1	1
KIAA0895	ENST00000297063.6	KIAA0895	1104	1	1	0
SLC25A21	ENST00000331299.5	solute carrier	110	1	1	0
TMEM182	ENST00000412401.2	transmembra	69	1	1	0
CCDC148	ENST00000283233.5	coiled-coil d	5	1	1	0
OTX2	ENST00000339475.5	orthodenticle	5	1	1	0
SDR42E1	ENST00000328945.5	short chain d	164	2	1	0
MYBL1	ENST00000522677.3	v-myb avian	53	1	0	1
RSPH10B	ENST00000539903.1	radial spoke	5	1	1	0
RNF111	ENST00000348370.4	ring finger pr	395	1	1	0
ZBTB10	ENST00000430430.1	zinc finger ar	113	2	1	0
AEBP2	ENST00000266508.9	AE binding p	1228	1	1	0
TLN2	ENST00000561311.1	talin 2	371	1	1	0
KCNQ3	ENST00000388996.4	potassium vo	5	1	1	0
BCAT1	ENST00000261192.7	branched cha	1346	1	1	0
ATF3	ENST00000366983.1	activating tra	2704	1	1	0
GATA2	ENST00000341105.2	GATA bindi	231	1	1	0
DRD5	ENST00000304374.2	dopamine rec	5	1	1	0
BMI1	ENST00000376663.3	BMI1 polyco	200	1	1	0
PLCXD3	ENST00000377801.3	phosphatidyl	81	1	0	1
TMEM33	ENST00000504986.1	transmembra	1470	2	1	0
FAM13C	ENST00000373867.3	family with s	343	1	1	0
CNOT8	ENST00000285896.6	CCR4-NOT	319	1	1	0
ZNF367	ENST00000375256.4	zinc finger pr	325	1	1	0
TGFBR1	ENST00000374994.4	transforming	249	2	0	1

PTPN12	ENST00000248594.6	protein tyros	167	1	1	0
BICC1	ENST00000373886.3	bicaudal C h	165	1	0	1
COMMD3-E	ENST00000602390.1	COMMD3-E	195	1	1	0
KCNRG	ENST00000360473.4	potassium ch	5	1	0	1
MAS1	ENST00000252660.4	MAS1 oncog	5	1	0	1
GRM7	ENST00000486284.1	glutamate rec	5	1	1	0
SLC19A2	ENST00000236137.5	solute carrier	229	1	0	1
RGL1	ENST00000304685.4	ral guanine n	188	1	1	0
ANKIB1	ENST00000265742.3	ankyrin repea	127	2	2	0
RASSF8	ENST00000541490.1	Ras associati	1290	1	1	0
PDSS1	ENST00000376203.5	prenyl (decap	41	1	0	1
MAP3K7	ENST00000369325.3	mitogen-activ	1172	3	0	1
TSC1	ENST00000298552.3	tuberous scle	838	1	1	0
BICD2	ENST00000356884.6	bicaudal D h	38	1	1	0
AC012215.1	ENST00000437887.1	Uncharacteri	31	1	0	0
PCNP	ENST00000296024.5	PEST proteo	944	2	1	0
FOXO3	ENST00000343882.6	forkhead box	32	2	1	1
FGB	ENST00000302068.4	fibrinogen be	1017	1	0	1
GRIK1-AS2	ENST00000333765.4	GRIK1 antis	5	1	0	0
FSTL4	ENST00000265342.7	follistatin-lik	60	1	1	0
CACNA2D1	ENST00000356860.3	calcium chan	418	1	1	0
DYX1C1	ENST00000457155.2	dyslexia susc	5	1	0	1
ONECUT2	ENST00000491143.2	one cut home	847	2	0	2
SEMA3C	ENST00000265361.3	sema domain	45	2	0	1
ZBTB16	ENST00000335953.4	zinc finger an	73	2	0	2
ZNF800	ENST00000393313.1	zinc finger pr	239	1	0	1
PHF21B	ENST00000403565.1	PHD finger p	8	1	1	0
MST4	ENST00000394334.2	Serine/threon	47	2	0	1
SALL3	ENST00000575389.2	sal-like 3 (Dr	64	2	1	1
NT5C3A	ENST00000610140.1	5'-nucleotida	165	1	0	1
CAV2	ENST00000222693.4	caveolin 2	1191	1	0	1
ECT2	ENST00000232458.5	epithelial cel	220	1	0	1
FMR1	ENST00000370471.3	fragile X me	1155	1	1	0
FCHO2	ENST00000430046.2	FCH domain	158	1	1	0
RASA2	ENST00000286364.3	RAS p21 pro	170	1	0	1
VPS54	ENST00000409558.4	vacuolar prot	312	1	1	0
HOXD3	ENST00000249440.3	homeobox D	23	1	1	0
SERPINF1	ENST00000254722.4	serpin peptid	596	1	0	1
CELF1	ENST00000395290.2	CUGBP, Ela	153	2	0	0
OTUD7A	ENST00000307050.4	OTU domain	35	1	1	0
PHTF2	ENST00000416283.2	putative hom	1730	1	1	0
TFRC	ENST00000540528.1	transferrin re	296	1	0	0
PAQR3	ENST00000512733.1	progestin and	217	2	1	0
ARFGEF1	ENST00000262215.3	ADP-ribosyla	70	2	2	0
IBTK	ENST00000306270.7	inhibitor of B	40	1	1	0
TMEM132B	ENST00000299308.3	transmembra	5	1	0	1
LHFP	ENST00000379589.3	lipoma HMG	5	1	0	1
PPFIA2	ENST00000549396.1	protein tyros	5	1	1	0

DACH1	ENST00000305425.4	dachshund ho	18	2	0	0
SLC32A1	ENST00000217420.1	solute carrier	5	1	0	0
USP6	ENST00000332776.4	ubiquitin spe	5	2	0	0
ZNF667	ENST00000591790.1	zinc finger pr	24	1	0	0
TXNDC9	ENST00000264255.3	thioredoxin c	873	1	0	0
HDHD2	ENST00000300605.6	haloacid deh	501	1	0	1
BRD3	ENST00000303407.7	bromodomai	77	1	0	1
ARHGAP12	ENST00000311380.4	Rho GTPase	190	2	0	1
CLCN6	ENST00000312413.6	chloride char	227	1	0	1
ADAMTS18	ENST00000282849.5	ADAM meta	7	1	1	0
FAM73B	ENST00000277475.5	family with s	651	1	0	1
FSTL5	ENST00000306100.5	follistatin-lik	5	1	0	1
PPFIA1	ENST00000253925.7	protein tyros	760	1	1	0
KIAA0247	ENST00000342745.4	KIAA0247	161	1	0	1
STAM	ENST00000377524.3	signal transd	723	1	0	0
PARD6B	ENST00000371610.2	par-6 family	277	1	0	1
ZC3H12A	ENST00000373087.6	zinc finger C	94	1	0	1
UST	ENST00000367463.4	uronyl-2-sulf	933	1	0	1
PYGO1	ENST00000302000.6	pygopus hom	104	1	0	1
E2F3	ENST00000346618.3	E2F transcrip	91	1	0	0
XKRX	ENST00000468904.1	XK, Kell blo	11	1	0	0
SREK1	ENST00000334121.6	splicing regu	316	2	2	0
ZNF385B	ENST00000410066.1	zinc finger pr	17	1	0	1
MINPP1	ENST00000371994.4	multiple inos	1079	1	0	1
CTNNB1	ENST00000349496.5	catenin (cadh	7257	1	0	1
BBX	ENST00000415149.2	bobby sox ho	141	2	0	2
PAXIP1	ENST00000404141.1	PAX interact	19	1	0	1
ZCCHC24	ENST00000372336.3	zinc finger, C	359	1	0	0
PRKG1	ENST00000373985.1	protein kinas	12	2	0	0
MET	ENST00000397752.3	met proto-on	1155	1	0	1
HMGN5	ENST00000358130.2	high mobility	46	1	1	0
MAP1LC3C	ENST00000357246.3	microtubule-	5	1	0	0
WASL	ENST00000223023.4	Wiskott-Aldr	230	1	1	0
PRKCA	ENST00000413366.3	protein kinas	726	1	0	0
HSPA4L	ENST00000296464.4	heat shock 70	348	2	0	1
RP6-24A23.(	ENST00000563887.1	Uncharacteri	937	1	0	0
HNRNPA3	ENST00000411529.2	heterogeneou	329	2	1	0
NPAS3	ENST00000346562.2	neuronal PAS	9	1	1	0
DCBLD2	ENST00000326840.6	discoidin, CU	798	1	0	1
CACHD1	ENST00000371073.2	cache domain	2071	1	0	1
EPN2	ENST00000314728.5	epsin 2	1609	1	0	1
ETV1	ENST00000430479.1	ets variant 1	160	2	0	1
SMARCA1	ENST00000371121.3	SWI/SNF rel	348	1	0	1
FAM19A1	ENST00000478136.1	family with s	5	1	0	0
SMIM13	ENST00000416247.2	small integra	199	1	0	1
STAU2	ENST00000522695.1	staufen doub	306	1	0	1
SKOR1	ENST00000341418.5	SKI family tr	22	1	0	0
ZNF749	ENST00000334181.4	zinc finger pr	69	1	0	1

PAFAH1B1	ENST00000397195.5	platelet-activ	1570	1	1	0
MACROD2	ENST00000217246.4	MACRO dor	5	1	0	1
ANKRD46	ENST00000335659.3	ankyrin repea	33	1	1	0
BEST1	ENST00000449131.2	bestrophin 1	83	1	0	0
SMO	ENST00000249373.3	smoothened,	142	1	0	1
PDE10A	ENST00000366882.1	phosphodiest	371	2	0	1
ALCAM	ENST00000306107.5	activated leu	371	1	0	1
SNAP91	ENST00000521485.1	synaptosoma	68	1	0	1
CDH2	ENST00000269141.3	cadherin 2, ty	1230	1	0	1
EIF2S3L	ENST00000538173.1	Putative euka	8	1	1	0
KITLG	ENST00000228280.5	KIT ligand	100	1	0	1
ASPN	ENST00000375543.1	asporin	5	1	0	0
B3GALT2	ENST00000367434.4	UDP-Gal:bet	5	1	0	0
PLEKHA1	ENST00000538022.1	pleckstrin ho	1172	1	0	0
RGS17	ENST00000367225.2	regulator of (	11	1	1	0
ROR2	ENST00000375708.3	receptor tyro	43	1	0	0
LAMA2	ENST00000421865.2	laminin, alph	5	1	0	0
IQCK	ENST00000320394.6	IQ motif con	42	1	0	1
TBC1D12	ENST00000225235.4	TBC1 domai	172	1	0	0
SELT	ENST00000471696.1	Selenoprotei	538	1	0	0
CPEB3	ENST00000412050.4	cytoplasmic p	48	2	0	0
KIF13A	ENST00000378814.5	kinesin famil	725	1	0	0
GNA13	ENST00000439174.2	guanine nucl	190	1	0	1
YWHAZ	ENST00000395957.2	tyrosine 3-mo	184	1	0	0
VAPB	ENST00000395802.3	VAMP (vesic	391	1	0	0
PLD1	ENST00000342215.6	phospholipas	128	2	0	1
HTRA1	ENST00000368984.3	HtrA serine p	1012	1	0	0
PICK1	ENST00000404072.3	protein intera	155	1	0	0
SYNPR	ENST00000479198.1	synaptoporin	5	1	0	1
MSRB3	ENST00000308259.5	methionine s	83	1	0	0
WDR44	ENST00000254029.3	WD repeat d	392	1	0	1
ARHGEF3	ENST00000296315.3	Rho guanine	359	1	0	1
CTGF	ENST00000367976.3	connective ti	10083	1	0	0
KLHL9	ENST00000359039.4	kelch-like fa	222	1	0	0
DTX1	ENST00000257600.3	deltex homol	10	1	0	0
KLHL13	ENST00000371882.1	kelch-like fa	13	1	0	0
FAM199X	ENST00000493442.1	family with s	856	1	0	1
EFNA5	ENST00000333274.6	ephrin-A5	225	1	0	0
PARPBP	ENST00000378128.3	PARP1 bindi	456	1	0	0
LRIG1	ENST00000273261.3	leucine-rich r	241	1	0	0
FLOT2	ENST00000394906.2	flotillin 2	37	2	0	1
TULP4	ENST00000367094.2	tubby like pr	93	1	0	0
CTNNAL1	ENST00000374595.4	catenin (cadh	5702	1	0	0
ZNF492	ENST00000456783.2	zinc finger pr	5	1	0	0
SRRM1	ENST00000323848.9	serine/argini	7	1	0	0
AXIN2	ENST00000307078.5	axin 2	86	1	0	0
MYB	ENST00000367814.4	v-myb avian	39	1	0	0
CAPRIN2	ENST00000395805.2	caprin family	17	1	0	1

USP7	ENST00000344836.4	ubiquitin spe	656	1	0	0
SOCS6	ENST00000397942.3	suppressor of	140	1	1	0
TP63	ENST00000392460.3	tumor protein	0	1	0	0
TMEM200A	ENST00000296978.3	transmembra	1609	1	0	0
BRWD3	ENST00000373275.4	bromodomain	506	1	1	0
HMGXB4	ENST00000216106.5	HMG box do	966	1	0	0
NUP54	ENST00000264883.3	nucleoporin 5	721	1	0	1
DDX42	ENST00000578681.1	DEAD (Asp-	797	1	0	0
PTGER4	ENST00000302472.3	prostaglandin	326	1	0	0
LONRF2	ENST00000393437.3	LON peptida	394	1	1	0
ZNF608	ENST00000504926.1	zinc finger pr	22	1	0	0
CACNB2	ENST00000396576.2	calcium chan	52	1	0	0
GJA1	ENST00000282561.3	gap junction	1732	1	0	0
C17orf103	ENST00000468196.1	chromosome	54	1	0	0
E2F7	ENST00000416496.2	E2F transcrip	308	1	0	0
SLC30A1	ENST00000367001.4	solute carrier	831	1	0	0
CD164	ENST00000368961.5	CD164 mole	2038	1	1	0
ATP2A2	ENST00000395494.2	ATPase, Ca+	2042	1	0	1
SRPK2	ENST00000393651.3	SRSF protein	381	1	0	1
TRERF1	ENST00000541110.1	transcription	52	1	0	0
STT3B	ENST00000295770.2	STT3B, subu	4458	1	0	0
PCDH10	ENST00000264360.5	protocadherin	9	1	0	0
MTSS1L	ENST00000338779.6	metastasis su	2982	1	0	0
NFIA	ENST00000403491.3	nuclear facto	5	3	1	0
BUB3	ENST00000368865.4	BUB3 mitoti	5237	1	0	0
WNT3	ENST00000225512.5	wingless-type	46	1	0	0
SAMSN1	ENST00000400564.1	SAM domain	5	1	0	0
SRSF6	ENST00000244020.3	serine/argini	1914	1	0	0
TEAD1	ENST00000361905.4	TEA domain	740	1	0	0
APPL1	ENST00000288266.3	adaptor prote	908	1	0	1
SLC25A13	ENST00000265631.5	solute carrier	8	1	0	1
SEMA6D	ENST00000355997.3	sema domain	179	1	0	1
KAT7	ENST00000259021.4	K(lysine) ace	48	1	0	0
PFN2	ENST00000239940.7	profilin 2	2184	1	0	0
CEP85L	ENST00000368491.3	centrosomal	14	1	0	0
STEAP2	ENST00000287908.3	STEAP fami	266	1	0	0
TSHZ1	ENST00000322038.5	teashirt zinc	174	1	0	0
UBR2	ENST00000372883.3	ubiquitin pro	728	1	0	0
SPRY3	ENST00000302805.2	sprouty homo	5	1	0	0
LUC7L3	ENST00000505658.1	LUC7-like 3	2970	1	0	0
ERN1	ENST00000433197.3	endoplasmic	182	1	0	0
AP3M1	ENST00000355264.4	adaptor-relat	601	1	0	0
STX16	ENST00000355957.5	syntaxin 16	756	1	0	0
BTBD1	ENST00000379403.2	BTB (POZ) d	84	1	1	0
SH3GL3	ENST00000535412.1	SH3-domain	74	1	0	1
MBTD1	ENST00000586178.1	mbt domain c	113	1	0	0
CADM2	ENST00000383699.3	cell adhesion	5	1	0	1
GOLPH3	ENST00000265070.6	golgi phosph	1157	1	0	0

CLUH	ENST00000570628.2	clustered mit	125	1	0	0
MYT1L	ENST00000399161.2	myelin transc	5	1	0	0
ITCH	ENST00000374864.4	itchy E3 ubic	769	1	1	0
MAPK8IP3	ENST00000250894.4	mitogen-activ	100	1	1	0
RNFT1	ENST00000442346.2	ring finger pı	6	1	0	0
ASAP2	ENST00000315273.4	ArfGAP with	38	1	1	0
CCDC144A	ENST00000443444.2	coiled-coil do	5	1	0	0
TMCC1	ENST00000432054.2	transmembra	97	1	0	1
RAB11FIP2	ENST00000355624.3	RAB11 famil	449	2	1	1
LYST	ENST00000389794.3	lysosomal tra	9	1	1	0
DR1	ENST00000370272.4	down-regulat	2994	2	0	0
PRDM6	ENST00000407847.4	PR domain c	69	1	0	0
SMARCA2	ENST00000349721.2	SWI/SNF rel	158	1	0	0
PBRM1	ENST00000356770.4	polybromo 1	689	1	0	0
C8orf34	ENST00000337103.4	chromosome	38	1	0	0
KLHL2	ENST00000538127.1	kelch-like fa	511	1	0	1
BCL9	ENST00000234739.3	B-cell CLL/l	70	1	0	0
ADAM22	ENST00000398204.4	ADAM meta	115	2	0	1
PTK6	ENST00000217185.2	protein tyros	27	1	0	1
ZBTB18	ENST00000358704.4	zinc finger ar	935	1	0	0
ZNF30	ENST00000601957.1	zinc finger pı	99	1	0	0
RASSF3	ENST00000542104.1	Ras associati	461	1	0	1
ZNF672	ENST00000306562.3	zinc finger pı	189	1	0	0
RNF38	ENST00000259605.6	ring finger pı	348	1	0	0
DOK6	ENST00000382713.5	docking prot	8	1	1	0
TENM2	ENST00000519204.1	teneurin tran	162	1	0	0
GLUD1	ENST00000277865.4	glutamate de	287	1	1	0
ERBB4	ENST00000342788.4	v-erb-b2 avia	27	1	0	0
LAMA5	ENST00000252999.3	laminin, alph	267	1	0	0
LHX9	ENST00000367390.3	LIM homeob	40	1	0	1
ANKRD28	ENST00000399451.2	ankyrin repea	798	1	0	0
ONECUT1	ENST00000560699.2	one cut home	10	2	0	1
PRTFDC1	ENST00000376378.1	phosphoribos	155	1	0	0
XPR1	ENST00000367590.4	xenotropic ar	544	1	0	0
KAT2B	ENST00000263754.4	K(lysine) ace	117	1	0	0
ZNF217	ENST00000371471.2	zinc finger pı	668	1	0	0
MTMR9	ENST00000221086.3	myotubularin	400	1	0	0
GRIK4	ENST00000527524.2	glutamate rec	5	1	0	0
TXNDC5	ENST00000539054.1	thioredoxin c	1190	1	0	1
STK39	ENST00000355999.4	serine threon	266	1	0	1
ARL4A	ENST00000396663.1	ADP-ribosyla	213	1	0	0
IGF1R	ENST00000268035.6	insulin-like g	561	2	0	1
ARL6IP5	ENST00000273258.3	ADP-ribosyla	150	1	0	0
MAPK14	ENST00000229795.3	mitogen-activ	1191	1	0	0
SLC38A2	ENST00000256689.5	solute carrier	696	1	0	0
ITGAV	ENST00000261023.3	integrin, alph	2407	1	0	0
PARD3B	ENST00000406610.2	par-3 family	38	1	0	0
HS2ST1	ENST00000370550.5	heparan sulfa	787	1	0	0

KLHL15	ENST00000328046.8	kelch-like fa	42	1	0	1
UGCG	ENST00000374279.3	UDP-glucose	1200	1	0	0
HOXD4	ENST00000306324.3	homeobox D	214	1	0	0
STK40	ENST00000359297.2	serine/threon	214	1	0	0
NAB1	ENST00000337386.5	NGFI-A binc	279	1	0	1
APBB2	ENST00000295974.8	amyloid beta	704	1	0	0
ZBTB20	ENST00000462705.1	zinc finger a	5	3	0	1
PAN3	ENST00000282391.5	PAN3 poly(A	361	1	0	1
ATXN7	ENST00000295900.6	ataxin 7	264	1	0	0
EGFR	ENST00000275493.2	epidermal gr	632	1	0	1
DLG5	ENST00000372391.2	discs, large h	10	1	0	0
C5orf24	ENST00000394976.3	chromosome	2668	1	0	0
IMPG1	ENST00000369963.3	interphotorec	5	1	0	0
PATZ1	ENST00000405309.3	POZ (BTB) a	165	1	0	1
G3BP2	ENST00000395719.3	GTPase activ	4413	1	0	0
MAFF	ENST00000338483.2	v-maf avian r	160	1	0	0
SORBS1	ENST00000371227.4	sorbin and S	22	1	0	0
TXLNG	ENST00000380122.5	taxilin gamm	96	1	0	0
NOS1AP	ENST00000361897.5	nitric oxide s	5	1	0	1
YPEL5	ENST00000379520.3	yippee-like 5	307	1	0	0
CLOCK	ENST00000309964.4	clock circadi	86	1	0	1
ATP13A3	ENST00000439040.1	ATPase type	243	1	0	1
HELZ	ENST00000358691.5	helicase with	571	1	0	0
TMTC3	ENST00000266712.6	transmembra	406	1	0	0
CUL5	ENST00000393094.2	cullin 5	761	1	0	0
RNF144A	ENST00000320892.6	ring finger pr	124	1	0	0
SLC7A14	ENST00000231706.5	solute carrier	5	1	0	1
YPEL2	ENST00000312655.4	yippee-like 2	62	1	0	0
INO80D	ENST00000403263.1	INO80 comp	5	1	0	0
CDYL	ENST00000343762.5	chromodoma	24	1	0	0
SAMD8	ENST00000372687.4	sterile alpha	100	1	0	1
RSF1	ENST00000308488.6	remodeling a	216	1	0	1
LMNB2	ENST00000325327.3	lamin B2	89	1	0	0
SPOPL	ENST00000280098.4	speckle-type	216	1	0	0
NEBL	ENST00000377122.4	nebulette	181	1	0	0
CNTN1	ENST00000551295.2	contactin 1	40	1	0	0
SLC44A5	ENST00000370859.3	solute carrier	395	1	0	0
SLC10A7	ENST00000264986.3	solute carrier	77	1	0	1
CDK14	ENST00000380050.3	cyclin-depen	226	1	0	0
RCAN3	ENST00000374395.4	RCAN famil	395	1	0	0
ZBTB33	ENST00000326624.2	zinc finger a	65	1	1	0
IRS2	ENST00000375856.3	insulin recep	231	1	0	1
NUTF2	ENST00000219169.4	nuclear trans	222	1	0	0
ZBTB2	ENST00000325144.4	zinc finger a	832	1	0	0
TFAP2A	ENST00000379613.3	transcription	367	1	0	1
CASP8	ENST00000392259.2	caspase 8, ap	64	1	0	0
SLC12A2	ENST00000262461.2	solute carrier	655	1	0	1
VAV3	ENST00000370056.4	vav 3 guanin	1128	1	0	1

NHSL1	ENST00000427025.2	NHS-like 1	393	1	0	0
MAP7D2	ENST00000379651.3	MAP7 doma	490	1	0	1
HEY1	ENST00000354724.3	hairy/enhanc	33	1	1	0
IRF2	ENST00000393593.3	interferon re	420	1	0	0
USP27X	ENST00000508866.2	ubiquitin spe	260	1	0	0
NF1	ENST00000358273.4	neurofibromi	161	1	1	0
UBE2E3	ENST00000410062.4	ubiquitin-cor	15	1	0	0
CXADR	ENST00000356275.6	coxsackie vi	56	1	1	0
ROBO1	ENST00000436010.2	roundabout,	546	1	0	0
SGIP1	ENST00000371036.3	SH3-domain	180	1	0	1
SNX18	ENST00000343017.6	sorting nexin	174	1	0	0
BCL2	ENST00000398117.1	B-cell CLL/l	55	1	0	0
PLXNA1	ENST00000393409.2	plexin A1	189	1	0	1
GRIA2	ENST00000296526.7	glutamate rec	5	1	0	0
LDLRAD4	ENST00000399848.3	low density l	314	1	0	0
CREBZF	ENST00000398294.2	CREB/ATF l	1115	1	0	0
PTPN3	ENST00000412145.1	protein tyros	374	1	1	0
NBR1	ENST00000542611.1	neighbor of I	1982	1	0	0
BIRC6	ENST00000421745.2	baculoviral I	555	1	0	0
PUM2	ENST00000338086.5	pumilio RNA	1047	1	0	0
USP22	ENST00000261497.4	ubiquitin spe	2152	1	0	0
ZFP1	ENST00000464850.1	ZFP1 zinc fi	32	1	0	1
ARRDC4	ENST00000268042.6	arrestin doma	10	1	0	0
PKN2	ENST00000370521.3	protein kinas	747	1	1	0
RAB8B	ENST00000321437.4	RAB8B, mer	45	1	0	0
SGCD	ENST00000435422.3	sarcoglycan,	39	1	0	0
ATF2	ENST00000487334.2	activating tra	123	1	1	0
MBD5	ENST00000407073.1	methyl-CpG	7	1	0	0
CDK19	ENST00000368911.3	cyclin-depen	51	2	0	1
MMP16	ENST00000286614.6	matrix metal	12	1	1	0
ZC3H12B	ENST00000338957.4	zinc finger C	29	1	0	1
SERTAD2	ENST00000313349.3	SERTA dom	119	1	0	1
HOMER1	ENST00000508576.1	homer homol	394	1	0	0
PPP3R1	ENST00000234310.3	protein phos	117	1	0	1
AP5M1	ENST00000261558.3	adaptor-relat	198	1	0	0
RBMS3	ENST00000396583.3	RNA binding	56	1	0	1
LDB1	ENST00000361198.5	LIM domain	61	1	0	1
LARP1B	ENST00000441387.1	La ribonucle	32	1	0	0
CHD7	ENST00000423902.2	chromodoma	925	1	1	0
OTUB1	ENST00000538426.1	OTU domain	2977	1	0	0
PALLD	ENST00000261509.6	palladin, cyt	4423	1	0	1
LMO4	ENST00000370544.5	LIM domain	294	1	0	0
SLC4A10	ENST00000415876.2	solute carrie	5	1	0	0
FBXO33	ENST00000298097.7	F-box protei	306	1	0	0
ARHGEF38	ENST00000420470.2	Rho guanine	5	1	0	0
EXOC5	ENST00000413566.2	exocyst com	242	1	0	0
FAM46A	ENST00000369754.3	family with s	899	1	0	0
UNC5C	ENST00000453304.1	unc-5 homol	60	1	0	0

NFATC3	ENST00000329524.4	nuclear facto	221	1	0	0
PPM1H	ENST00000228705.6	protein phos	45	1	1	0
HGF	ENST00000222390.5	hepatocyte g	18	1	0	1
GJC1	ENST00000426548.1	gap junction	195	1	1	0
DUSP16	ENST00000298573.4	dual specific	444	2	0	1
MPDZ	ENST00000319217.7	multiple PDZ	341	1	0	1
GSKIP	ENST00000555181.1	GSK3B inter	1581	1	0	0
NOVA1	ENST00000465357.2	neuro-oncolo	5	1	0	0
DLG2	ENST00000398309.2	discs, large h	15	1	0	0
DCUN1D1	ENST00000292782.4	DCN1, defec	267	1	0	0
CNKSR2	ENST00000379510.3	connector en	85	1	0	1
FOXP1	ENST00000318789.4	forkhead box	264	2	1	0
UBN1	ENST00000262376.6	ubinuclein 1	586	1	0	1
TANC2	ENST00000424789.2	tetratricopep	155	1	0	0
MTM1	ENST00000542741.1	myotubularin	68	1	0	0
PER2	ENST00000254658.3	period circad	106	1	0	0
AFF2	ENST00000370460.2	AF4/FMR2 f	8	1	0	0
PTBP3	ENST00000374257.1	polypyrimidi	214	2	0	1
CDYL2	ENST00000570137.2	chromodoma	239	1	0	0
TNRC6C	ENST00000335749.4	trinucleotide	248	1	0	0
WWC2	ENST00000403733.3	WW and C2	717	1	0	0
CXorf23	ENST00000379687.3	chromosome	30	1	1	0
IQSEC1	ENST00000273221.4	IQ motif and	415	1	0	0
LMTK2	ENST00000297293.5	lemur tyrosin	51	2	0	1
OTUD6B	ENST00000285420.4	OTU domain	18	1	1	0
BCORL1	ENST00000540052.1	BCL6 corepr	209	1	1	0
ZDHHC17	ENST00000426126.2	zinc finger, I	117	1	0	0
KCNB1	ENST00000371741.4	potassium vo	5	2	0	1
UNC5D	ENST00000287272.2	unc-5 homolo	37	1	0	0
SYNC	ENST00000409190.3	syncoilin, int	353	1	0	0
EGFLAM	ENST00000322350.5	EGF-like, fib	123	1	0	0
AKAP13	ENST00000394518.2	A kinase (PR	53	1	0	1
ZC3H12C	ENST00000278590.3	zinc finger C	150	1	0	0
EYA4	ENST00000367895.5	eyes absent h	242	1	0	0
TWIST1	ENST00000242261.5	twist basic he	190	1	0	0
ABCC5	ENST00000334444.6	ATP-binding	453	1	0	1
SUZ12	ENST00000322652.5	SUZ12 polyc	104	2	1	0
PIK3CA	ENST00000263967.3	phosphatidyl	112	1	0	0
RPUSD1	ENST00000565809.1	RNA pseudo	1360	1	0	0
STAG2	ENST00000371160.1	stromal antig	216	1	0	0
FNDC3B	ENST00000336824.4	fibronectin ty	509	1	0	0
RFX3	ENST00000382004.3	regulatory fa	37	1	0	0
MOB4	ENST00000233892.4	MOB family	123	2	0	1
NUCKS1	ENST00000367142.4	nuclear casei	1692	1	0	1
ZFHX4	ENST00000521891.2	zinc finger ho	194	1	1	0
SLC7A1	ENST00000380752.5	solute carrier	338	1	0	0
PHF20	ENST00000439301.1	PHD finger p	191	1	0	0
JHDM1D	ENST00000397560.2	jumonji C do	470	1	0	0

VCL	ENST00000372755.3	vinculin	189	1	0	1
CCDC88A	ENST00000336838.6	coiled-coil do	141	1	0	0
RORA	ENST00000335670.6	RAR-related	102	1	0	0
GPM6B	ENST00000454189.2	glycoprotein	135	1	0	0
HMGB1	ENST00000399489.1	high mobility	800	1	0	0
BRD1	ENST00000457780.2	bromodomain	124	1	1	0
PRLR	ENST00000342362.5	prolactin rece	95	1	0	1
WNT5A	ENST00000474267.1	wingless-type	2080	1	0	0
CAV1	ENST00000405348.1	caveolin 1, ca	415	1	0	0
SZRD1	ENST00000401089.3	SUZ RNA bi	137	1	0	1
MRRF	ENST00000344641.3	mitochondria	85	1	0	0
POU2F1	ENST00000367866.2	POU class 2	83	1	0	0
GNAL	ENST00000334049.6	guanine nucl	99	1	0	0
UBA6	ENST00000322244.5	ubiquitin-like	169	1	0	1
CREB5	ENST00000357727.2	cAMP respon	54	1	0	0
COBL	ENST00000265136.7	cordon-bleu	205	1	0	0
PLAG1	ENST00000316981.3	pleiomorphic	58	1	0	0
ETNK1	ENST00000266517.4	ethanolamine	1047	1	0	1
PPM1E	ENST00000308249.2	protein phosp	178	1	0	0
PHYHIPL	ENST00000373880.4	phytanoyl-Co	26	1	0	0
HSPE1-MOB	ENST00000604458.1	HSPE1-MOB	123	2	0	1
PHC3	ENST00000495893.2	polyhomeotic	366	1	0	0
MARK1	ENST00000366918.4	MAP/microtu	192	1	0	0
ATXN1	ENST00000244769.4	ataxin 1	106	2	0	0
FUBP1	ENST00000370767.1	far upstream	2021	1	0	0
MKX	ENST00000375790.5	mohawk hom	165	1	0	1
SLC38A4	ENST00000447411.1	solute carrier	10	1	0	0
CUX2	ENST00000261726.6	cut-like home	15	1	1	0
CTDSPL2	ENST00000260327.4	CTD (carbox	457	1	0	0
FAM171B	ENST00000304698.5	family with s	5	1	0	0
TBL1XR1	ENST00000430069.1	transducin (b	334	2	0	0
TNRC6B	ENST00000335727.9	trinucleotide	69	2	0	1
TNPO1	ENST00000337273.5	transportin 1	2041	1	0	0
SOX5	ENST00000546136.1	SRY (sex det	43	1	0	1
DLC1	ENST00000276297.4	deleted in liv	970	1	0	0
CHD2	ENST00000394196.4	chromodoma	1237	1	0	0
XKR4	ENST00000327381.6	XK, Kell blo	5	1	0	0
TRPM3	ENST00000377110.3	transient rece	11	1	0	1
ZNF148	ENST00000360647.4	zinc finger pr	230	1	0	1
MED13L	ENST00000281928.3	mediator com	73	1	0	0
MON2	ENST00000546600.1	MON2 homo	784	2	1	1
CMTM4	ENST00000394106.2	CKLF-like M	22	1	0	0
ZNF439	ENST00000304030.2	zinc finger pr	64	1	0	0
ATAD2B	ENST00000238789.5	ATPase fami	11	1	0	0
EIF2S1	ENST00000256383.4	eukaryotic tra	3360	1	0	1
STRBP	ENST00000447404.2	spermatid pe	575	1	1	0
ATP1B3	ENST00000539728.1	ATPase, Na+	269	1	0	1
B3GALT1	ENST00000392690.3	UDP-Gal:bet	35	1	0	0

NRXN1	ENST00000342183.5	neurexin 1	5	1	0	0
MARCH5	ENST00000358935.2	membrane-as	881	1	0	1
BTF3L4	ENST00000489308.2	basic transcri	1245	2	0	0
SLC8A1	ENST00000406785.2	solute carrier	61	2	1	1
SOS1	ENST00000426016.1	son of sevenl	189	1	0	0
USP16	ENST00000399975.3	ubiquitin spe	42	1	0	0
USP25	ENST00000285681.2	ubiquitin spe	2012	2	0	2
HOOK3	ENST00000307602.4	hook microtu	403	1	0	1
HHIP	ENST00000296575.3	hedgehog int	65	1	0	1
YAF2	ENST00000327791.4	YY1 associat	413	1	0	0
NOTCH1	ENST00000277541.6	notch 1	88	1	0	1
EPHA5	ENST00000273854.3	EPH receptor	48	1	0	0
GEMIN6	ENST00000409011.1	gem (nuclear	257	1	0	0
FAF1	ENST00000396153.2	Fas (TNFRS	428	1	0	0
FAM49A	ENST00000381323.3	family with s	38	1	0	0
WDR41	ENST00000296679.4	WD repeat d	313	1	0	0
RHOQ	ENST00000238738.4	ras homolog	179	1	0	0
GUCY1A3	ENST00000296518.7	guanylate cy	42	1	0	0
ZNF138	ENST00000440598.1	zinc finger p	357	1	0	0
SLC24A3	ENST00000328041.6	solute carrier	63	1	1	0
NETO2	ENST00000562435.1	neuropilin (N	723	1	1	0
FAM126A	ENST00000409923.1	family with s	241	1	0	0
MRAS	ENST00000289104.4	muscle RAS	181	1	0	0
PITPNC1	ENST00000580974.1	phosphatidyl	797	1	1	0
CBLB	ENST00000264122.4	Cbl proto-on	469	1	0	0
APPBP2	ENST00000083182.3	amyloid beta	907	1	1	0
GAB1	ENST00000262995.4	GRB2-associ	26	1	0	0
MTUS2	ENST00000431530.3	microtubule	17	1	0	0
SLC40A1	ENST00000261024.2	solute carrier	25	1	0	0
ZDHHC21	ENST00000380916.4	zinc finger, I	196	1	0	1
DOCK11	ENST00000276204.6	dedicator of	40	1	0	1
PACS2	ENST00000447393.1	phosphofurin	234	1	0	1
MBNL3	ENST00000370839.3	muscleblind-	593	1	0	0
NCAM1	ENST00000316851.7	neural cell ac	19	1	0	0
EPC1	ENST00000319778.6	enhancer of p	102	1	0	1
TP53INP1	ENST00000448464.2	tumor protein	39	1	0	0
IRF2BP2	ENST00000366610.3	interferon reg	8690	1	0	0
NUP50	ENST00000347635.4	nucleoporin 5	57	1	0	1
USP51	ENST00000500968.3	ubiquitin spe	26	1	0	0
MSL2	ENST00000309993.2	male-specific	175	2	0	1
PCYOX1	ENST00000264441.5	prenylcystein	407	1	0	0
SIN3A	ENST00000394947.3	SIN3 transcri	1142	1	0	0
LGALSL	ENST00000409537.2	lectin, galact	334	1	0	0
VGLL4	ENST00000273038.3	vestigial like	37	2	0	0
MSH6	ENST00000234420.5	mutS homolo	94	1	1	0
KCNQ5	ENST00000370398.1	potassium vo	50	1	0	1
STMN2	ENST00000518111.1	stathmin-like	7	1	0	1
SMURF2	ENST00000262435.9	SMAD speci	978	1	0	0

CUL4B	ENST00000371322.5	cullin 4B	150	1	0	1
FAM49B	ENST00000519824.2	family with s	52	1	0	1
MYEF2	ENST00000324324.7	myelin expre	358	1	0	0
MESDC1	ENST00000267984.2	mesoderm de	84	1	0	0
SNX13	ENST00000409389.1	sorting nexin	100	1	0	0
GIGYF2	ENST00000373566.3	GRB10 inter	361	1	0	1
CFL2	ENST00000341223.3	cofilin 2 (mu	902	1	0	0
MLLT3	ENST00000380338.4	myeloid/lym	385	1	0	1
BICD1	ENST00000548411.1	bicaudal D h	205	1	0	1
ALG6	ENST00000371108.4	ALG6, alpha	140	1	0	0
MAP2	ENST00000360351.4	microtubule-	68	1	0	1
RAB3C	ENST00000282878.4	RAB3C, mer	242	1	0	0
CNKSR3	ENST00000607772.1	CNKSR fam	567	1	0	0
CDH4	ENST00000360469.5	cadherin 4, t	33	1	0	0
MAGI1	ENST00000330909.8	membrane as	258	1	0	1
IPPK	ENST00000287996.3	inositol 1,3,4	211	1	0	0
ARHGAP19	ENST00000453547.2	ARHGAP19	15	1	0	0
PTPDC1	ENST00000375360.3	protein tyros	46	1	0	1
RSBN1	ENST00000261441.5	round sperma	54	1	0	0
DENND5A	ENST00000530044.1	DENN/MAD	294	1	0	0
VANGL1	ENST00000355485.2	VANGL plan	721	1	0	0
RRAGD	ENST00000369415.4	Ras-related C	644	1	0	0
PCSK9	ENST00000302118.5	proprotein co	2345	1	0	0
ACVR2A	ENST00000241416.7	activin A rec	109	1	0	0
GATA6	ENST00000269216.3	GATA bindi	691	2	0	1
NUP43	ENST00000367403.3	nucleoporin 4	113	1	0	1
SZT2	ENST00000562955.1	seizure thresh	123	1	0	0
PRPF40A	ENST00000410080.1	PRP40 pre-m	459	1	0	0
AGO3	ENST00000373191.4	argonaute RI	198	1	0	1
NR3C2	ENST00000344721.4	nuclear recep	425	1	0	0
PHF21A	ENST00000257821.4	PHD finger p	301	1	0	0
DICER1	ENST00000541352.1	dicer 1, ribon	25	1	0	0
DMXL1	ENST00000311085.8	Dmx-like 1	136	1	0	0
MTDH	ENST00000336273.3	metadherin	5192	1	0	1
IGSF10	ENST00000282466.3	immunoglobu	191	1	0	0
AGPS	ENST00000264167.4	alkylglyceron	1610	2	0	1
ELAVL1	ENST00000407627.2	ELAV like R	2812	1	0	0
STK24	ENST00000397517.2	serine/threon	486	1	0	1
CLCC1	ENST00000356970.2	chloride chan	849	1	0	0
HIC2	ENST00000407464.2	hypermethyla	28	1	0	0
CDC37L1	ENST00000381854.3	cell division	280	1	0	0
RLIM	ENST00000332687.6	ring finger pr	662	1	0	1
GRK5	ENST00000392870.2	G protein-cou	337	1	0	0
HMBOX1	ENST00000397358.3	homeobox co	27	1	0	0
HMGB3	ENST00000325307.7	high mobility	295	1	0	0
HIF1A	ENST00000323441.6	hypoxia indu	1955	1	0	0
MAP7	ENST00000354570.3	microtubule-	444	1	0	1
ATP2B2	ENST00000352432.4	ATPase, Ca+	170	1	0	0

ELOVL6	ENST00000394607.3	ELOVL fatty	901	1	1	0
ERO1LB	ENST00000354619.5	ERO1-like b	478	1	0	0
MED28	ENST00000237380.7	mediator con	1483	1	0	0
PDCD10	ENST00000392750.2	programmed	638	1	0	0
MELK	ENST00000536987.1	maternal emb	5978	2	0	1
RAB11A	ENST00000569896.1	RAB11A, me	5781	1	0	0
HPS1	ENST00000361490.4	Hermansky-P	382	1	0	0
HCN1	ENST00000303230.4	hyperpolariza	5	2	2	0
ZNF281	ENST00000294740.3	zinc finger pr	595	1	0	0
RNF11	ENST00000242719.3	ring finger pr	5881	1	0	1
HMGCR	ENST00000287936.4	3-hydroxy-3-	1385	1	0	0
KLF10	ENST00000285407.6	Kruppel-like	320	1	0	0
FGF10	ENST00000264664.4	fibroblast gro	5	2	0	1
RSU1	ENST00000377921.3	Ras suppress	10596	1	0	1
SCAF8	ENST00000367178.3	SR-related C	1917	1	0	0
TAB3	ENST00000378933.1	TGF-beta act	201	1	0	1
BCL9L	ENST00000334801.3	B-cell CLL/l	91	1	0	0
SRSF3	ENST00000373715.6	serine/argini	2593	1	1	0
CLEC14A	ENST00000342213.2	C-type lectin	5	1	0	0
CACNG2	ENST00000300105.6	calcium chan	5	1	0	0
CHST1	ENST00000308064.2	carbohydrate	5	1	0	0
CSMD1	ENST00000400186.3	CUB and Su	5	1	0	0
KDM6A	ENST00000377967.4	lysine (K)-sp	991	1	0	1
MAPK10	ENST00000395169.3	mitogen-acti	5	1	0	1
PIK3R5	ENST00000447110.1	phosphoinosi	5	1	0	0
MYBPC1	ENST00000392934.3	myosin bindi	5	1	0	0
ABI2	ENST00000295851.5	abl-interactor	877	1	0	0
PTPRF	ENST00000372414.3	protein tyros	14842	1	0	1
C16orf45	ENST00000300006.4	chromosome	60	1	1	0
ANKS1B	ENST00000546960.1	ankyrin repea	21	1	0	1
MXD4	ENST00000337190.2	MAX dimeri	1173	1	1	0
NAA50	ENST00000240922.3	N(alpha)-ace	260	1	0	0
VWA1	ENST00000338660.5	von Willebra	145	1	0	0
LMF1	ENST00000262301.11	lipase matura	155	1	0	0
ITGB1BP1	ENST00000360635.3	integrin beta	14310	1	0	0
RP11-73M1	ENST00000472726.2	Kinesin light	499	2	0	1
BMF	ENST00000220446.4	Bcl2 modifyi	179	1	0	0
DOCK7	ENST00000251157.5	dedicator of	66	1	0	1
RSPO3	ENST00000356698.4	R-spondin 3	118	1	0	0
DONSON	ENST00000453626.1	downstream	103	1	1	0
ATP6V1C1	ENST00000395862.3	ATPase, H+	951	1	0	0
SV2C	ENST00000502798.2	synaptic vesi	30	1	0	0
ROCK1	ENST00000399799.2	Rho-associat	7	1*	0	0
SESTD1	ENST00000428443.3	SEC14 and s	478	1*	0	0
PCDHB16	ENST00000361016.2	protocadheri	36	1*	0	0
KIF5B	ENST00000302418.4	kinesin famil	635	1*	0	0
GPR19	ENST00000540510.1	G protein-co	12	1*	0	0
KIF3B	ENST00000375712.3	kinesin famil	182	1*	0	0

FMNL3	ENST00000335154.5	formin-like 3	132	1*	0	0

Table S2 miR-458b-5p target genes predicted using miRDB				
**Rank**	**Score**	**miRNA name**	**Gene symbol**	**Gene description**
1	98	gga-miR-458b-5	RASA2	RAS p21 protein activator 2
2	97	gga-miR-458b-5	STK26	serine/threonine kinase 26
3	96	gga-miR-458b-5	MON2	MON2 homolog, regulator of endosome-to-Golgi traffickir
4	96	gga-miR-458b-5	LNPK	lunapark, ER junction formation factor
5	96	gga-miR-458b-5	STK24	serine/threonine kinase 24
6	95	gga-miR-458b-5	CLIC4	chloride intracellular channel 4
7	94	gga-miR-458b-5	ZBTB10	zinc finger and BTB domain containing 10
8	94	gga-miR-458b-5	CTNNB1	catenin beta 1
9	94	gga-miR-458b-5	ARFGEF1	ADP ribosylation factor guanine nucleotide exchange facto
10	94	gga-miR-458b-5	BCL9	B-cell CLL/lymphoma 9
11	94	gga-miR-458b-5	PHIP	pleckstrin homology domain interacting protein
12	93	gga-miR-458b-5	DPEP2	dipeptidase 2
13	93	gga-miR-458b-5	RAB11FIP2	RAB11 family interacting protein 2
14	93	gga-miR-458b-5	BICC1	BicC family RNA binding protein 1
15	92	gga-miR-458b-5	BRD1	bromodomain containing 1
16	92	gga-miR-458b-5	RNF111	ring finger protein 111
17	92	gga-miR-458b-5	TXLNG	taxilin gamma
18	92	gga-miR-458b-5	CACHD1	cache domain containing 1
19	92	gga-miR-458b-5	ATP1B3	ATPase Na+/K+ transporting subunit beta 3
20	91	gga-miR-458b-5	PHTF2	putative homeodomain transcription factor 2
21	91	gga-miR-458b-5	ITPRIPL2	inositol 1,4,5-trisphosphate receptor interacting protein like
22	90	gga-miR-458b-5	SRSF3	serine and arginine rich splicing factor 3
23	90	gga-miR-458b-5	FAM49B	family with sequence similarity 49 member B
24	90	gga-miR-458b-5	AHSA2P	activator of HSP90 ATPase homolog 2, pseudogene
25	90	gga-miR-458b-5	SRGAP3	SLIT-ROBO Rho GTPase activating protein 3
26	90	gga-miR-458b-5	PTPN12	protein tyrosine phosphatase, non-receptor type 12
27	89	gga-miR-458b-5	CARF	calcium responsive transcription factor
28	89	gga-miR-458b-5	DPYSL3	dihydropyrimidinase like 3
29	89	gga-miR-458b-5	UFSP2	UFM1 specific peptidase 2
30	89	gga-miR-458b-5	SUZ12	SUZ12, polycomb repressive complex 2 subunit
31	89	gga-miR-458b-5	TBX4	T-box 4
32	89	gga-miR-458b-5	VPS54	VPS54, GARP complex subunit
33	88	gga-miR-458b-5	UPB1	beta-ureidopropionase 1
34	88	gga-miR-458b-5	GAB2	GRB2 associated binding protein 2
35	88	gga-miR-458b-5	GRIK2	glutamate ionotropic receptor kainate type subunit 2
36	88	gga-miR-458b-5	SMAD5	SMAD family member 5
37	88	gga-miR-458b-5	STRBP	spermatid perinuclear RNA binding protein
38	88	gga-miR-458b-5	FOXO3	forkhead box O3
39	88	gga-miR-458b-5	TRAF3	TNF receptor associated factor 3
40	88	gga-miR-458b-5	OSBP2	oxysterol binding protein 2
41	87	gga-miR-458b-5	CDH2	cadherin 2
42	87	gga-miR-458b-5	SNAP91	synaptosome associated protein 91
43	87	gga-miR-458b-5	PARD3	par-3 family cell polarity regulator
44	86	gga-miR-458b-5	TOMM34	translocase of outer mitochondrial membrane 34
45	86	gga-miR-458b-5	KLHL23	kelch like family member 23
46	86	gga-miR-458b-5	PDPK1	3-phosphoinositide dependent protein kinase 1

47	85	gga-miR-458b-5	FAM102B	family with sequence similarity 102 member B
48	85	gga-miR-458b-5	ELOVL6	ELOVL fatty acid elongase 6
49	85	gga-miR-458b-5	ANKS1B	ankyrin repeat and sterile alpha motif domain containing 1[illegible]
50	85	gga-miR-458b-5	PSMD6	proteasome 26S subunit, non-ATPase 6
51	85	gga-miR-458b-5	AUH	AU RNA binding methylglutaconyl-CoA hydratase
52	84	gga-miR-458b-5	ECT2	epithelial cell transforming 2
53	84	gga-miR-458b-5	BAG1	BCL2 associated athanogene 1
54	84	gga-miR-458b-5	LHX9	LIM homeobox 9
55	84	gga-miR-458b-5	GNA13	G protein subunit alpha 13
56	83	gga-miR-458b-5	OTX2	orthodenticle homeobox 2
57	83	gga-miR-458b-5	MLEC	malectin
58	83	gga-miR-458b-5	ZNF367	zinc finger protein 367
59	83	gga-miR-458b-5	KLHL34	kelch like family member 34
60	83	gga-miR-458b-5	NF1	neurofibromin 1
61	83	gga-miR-458b-5	MESD	mesoderm development LRP chaperone
62	82	gga-miR-458b-5	GRM7	glutamate metabotropic receptor 7
63	82	gga-miR-458b-5	FMR1	fragile X mental retardation 1
64	82	gga-miR-458b-5	ANKIB1	ankyrin repeat and IBR domain containing 1
65	82	gga-miR-458b-5	CLTCL1	clathrin, heavy chain-like 1
66	82	gga-miR-458b-5	UBA6	ubiquitin like modifier activating enzyme 6
67	82	gga-miR-458b-5	CERS5	ceramide synthase 5
68	82	gga-miR-458b-5	CNOT8	CCR4-NOT transcription complex subunit 8
69	81	gga-miR-458b-5	DNTT	DNA nucleotidylexotransferase
70	81	gga-miR-458b-5	PPM1E	protein phosphatase, Mg2+/Mn2+ dependent 1E
71	80	gga-miR-458b-5	NT5C2	5'-nucleotidase, cytosolic II
72	80	gga-miR-458b-5	LMINA	lamin-L(III)-like
73	80	gga-miR-458b-5	ARL8B	ADP ribosylation factor like GTPase 8B
74	79	gga-miR-458b-5	EIF4E	eukaryotic translation initiation factor 4E
75	79	gga-miR-458b-5	HSPA4L	heat shock protein family A (Hsp70) member 4 like
76	79	gga-miR-458b-5	BCORL1	BCL6 corepressor like 1
77	78	gga-miR-458b-5	SH3D19	SH3 domain containing 19
78	78	gga-miR-458b-5	IPPK	inositol-pentakisphosphate 2-kinase
79	78	gga-miR-458b-5	STK39	serine/threonine kinase 39
80	77	gga-miR-458b-5	PPFIA1	PTPRF interacting protein alpha 1
81	77	gga-miR-458b-5	RDH7L	retinol dehydrogenase 7-like
82	77	gga-miR-458b-5	SMARCA1	SWI/SNF related, matrix associated, actin dependent regul
83	77	gga-miR-458b-5	AHSG	alpha 2-HS glycoprotein
84	77	gga-miR-458b-5	CDCA2	cell division cycle associated 2
85	77	gga-miR-458b-5	ADAMTS6	ADAM metallopeptidase with thrombospondin type 1 moti
86	77	gga-miR-458b-5	P4HA1	prolyl 4-hydroxylase subunit alpha 1
87	76	gga-miR-458b-5	BBX	BBX, HMG-box containing
88	76	gga-miR-458b-5	TTC21B	tetratricopeptide repeat domain 21B
89	76	gga-miR-458b-5	TBL1XR1	transducin beta like 1 X-linked receptor 1
90	76	gga-miR-458b-5	BEND6	BEN domain containing 6
91	76	gga-miR-458b-5	MYT1L	myelin transcription factor 1 like
92	76	gga-miR-458b-5	GUCY2F	guanylate cyclase 2F, retinal
93	76	gga-miR-458b-5	SLITRK6	SLIT and NTRK like family member 6
94	76	gga-miR-458b-5	APOBEC2	apolipoprotein B mRNA editing enzyme catalytic subunit [illegible]

95	76	gga-miR-458b-5	LDLRAD4	low density lipoprotein receptor class A domain containing
96	75	gga-miR-458b-5	DPT	dermatopontin
97	75	gga-miR-458b-5	PCDH10	protocadherin 10
98	75	gga-miR-458b-5	REV3L	REV3 like, DNA directed polymerase zeta catalytic subuni
99	75	gga-miR-458b-5	GID8	GID complex subunit 8 homolog
100	75	gga-miR-458b-5	ANKRD44	ankyrin repeat domain 44
101	75	gga-miR-458b-5	BRD3	bromodomain containing 3
102	74	gga-miR-458b-5	FAIM	Fas apoptotic inhibitory molecule
103	74	gga-miR-458b-5	PTPRF	protein tyrosine phosphatase, receptor type F
104	74	gga-miR-458b-5	RASSF2	Ras association domain family member 2
105	74	gga-miR-458b-5	LAMA5	laminin subunit alpha 5
106	74	gga-miR-458b-5	SLF2	SMC5-SMC6 complex localization factor 2
107	73	gga-miR-458b-5	DCBLD2	discoidin, CUB and LCCL domain containing 2
108	73	gga-miR-458b-5	SUSD6	sushi domain containing 6
109	73	gga-miR-458b-5	TWSG1	twisted gastrulation BMP signaling modulator 1
110	73	gga-miR-458b-5	LOC422051	zinc finger protein 91-like
111	73	gga-miR-458b-5	KLHL20	kelch like family member 20
112	73	gga-miR-458b-5	ARHGAP12	Rho GTPase activating protein 12
113	72	gga-miR-458b-5	FCHO2	FCH domain only 2
114	72	gga-miR-458b-5	HMGN5	high mobility group nucleosome binding domain 5
115	72	gga-miR-458b-5	FBXO45	F-box protein 45
116	72	gga-miR-458b-5	BRWD3	bromodomain and WD repeat domain containing 3
117	72	gga-miR-458b-5	SCNN1D	sodium channel epithelial 1 delta subunit
118	72	gga-miR-458b-5	ABCC3	ATP binding cassette subfamily C member 3
119	72	gga-miR-458b-5	ZNF512	zinc finger protein 512
120	72	gga-miR-458b-5	RASA3	RAS p21 protein activator 3
121	72	gga-miR-458b-5	SMYD3	SET and MYND domain containing 3
122	71	gga-miR-458b-5	VCL	vinculin
123	71	gga-miR-458b-5	DENND4A	DENN domain containing 4A
124	71	gga-miR-458b-5	PHF21B	PHD finger protein 21B
125	71	gga-miR-458b-5	DR1	down-regulator of transcription 1
126	70	gga-miR-458b-5	STT3B	STT3B, catalytic subunit of the oligosaccharyltransferase c
127	70	gga-miR-458b-5	LOC420294	cylindromatosis-like
128	70	gga-miR-458b-5	DIAPH2	diaphanous related formin 2
129	70	gga-miR-458b-5	ZBTB38	zinc finger and BTB domain containing 38
130	70	gga-miR-458b-5	SOWAHC	sosondowah ankyrin repeat domain family member C
131	69	gga-miR-458b-5	XYLT1	xylosyltransferase 1
132	69	gga-miR-458b-5	CDR2	cerebellar degeneration related protein 2
133	69	gga-miR-458b-5	PLCXD3	phosphatidylinositol specific phospholipase C X domain co
134	69	gga-miR-458b-5	KDM1B	lysine demethylase 1B
135	69	gga-miR-458b-5	COG3	component of oligomeric golgi complex 3
136	69	gga-miR-458b-5	PALLD	palladin, cytoskeletal associated protein
137	69	gga-miR-458b-5	SYTL3	synaptotagmin like 3
138	69	gga-miR-458b-5	C4HCXorf57	chromosome 4 CXorf57 homolog
139	69	gga-miR-458b-5	SREK1	splicing regulatory glutamic acid and lysine rich protein 1
140	69	gga-miR-458b-5	ARL4A	ADP ribosylation factor like GTPase 4A
141	68	gga-miR-458b-5	TMEM263	transmembrane protein 263
142	68	gga-miR-458b-5	PIK3C2G	phosphatidylinositol-4-phosphate 3-kinase catalytic subuni

143	68	gga-miR-458b-5	MET	MET proto-oncogene, receptor tyrosine kinase
144	68	gga-miR-458b-5	TNPO1	transportin 1
145	68	gga-miR-458b-5	FAM49A	family with sequence similarity 49 member A
146	68	gga-miR-458b-5	EPHX1	epoxide hydrolase 1
147	68	gga-miR-458b-5	POU2F1	POU class 2 homeobox 1
148	68	gga-miR-458b-5	TSC1	TSC complex subunit 1
149	68	gga-miR-458b-5	SNX13	sorting nexin 13
150	67	gga-miR-458b-5	LIN28B	lin-28 homolog B
151	67	gga-miR-458b-5	SLC9A3	solute carrier family 9 member A3
152	67	gga-miR-458b-5	NOVA1	uncharacterized LOC423300
153	67	gga-miR-458b-5	FREM3	FRAS1 related extracellular matrix 3
154	67	gga-miR-458b-5	GDE1	glycerophosphodiester phosphodiesterase 1
155	67	gga-miR-458b-5	IMPG2	interphotoreceptor matrix proteoglycan 2
156	67	gga-miR-458b-5	ALCAM	activated leukocyte cell adhesion molecule
157	66	gga-miR-458b-5	NUBP2	nucleotide binding protein 2
158	66	gga-miR-458b-5	SPRY3	sprouty RTK signaling antagonist 3
159	66	gga-miR-458b-5	C9ORF152	chromosome 2 open reading frame, human C9orf152
160	66	gga-miR-458b-5	MCMBP	minichromosome maintenance complex binding protein
161	66	gga-miR-458b-5	SMARCA2	SWI/SNF related, matrix associated, actin dependent regul
162	66	gga-miR-458b-5	DOCK6	dedicator of cytokinesis 6
163	66	gga-miR-458b-5	CUL4B	cullin 4B
164	66	gga-miR-458b-5	CECR5	cat eye syndrome chromosome region, candidate 5
165	66	gga-miR-458b-5	BIRC6	baculoviral IAP repeat containing 6
166	66	gga-miR-458b-5	CEBPD	CCAAT enhancer binding protein delta
167	65	gga-miR-458b-5	PITPNC1	phosphatidylinositol transfer protein, cytoplasmic 1
168	65	gga-miR-458b-5	KIF13A	kinesin family member 13A
169	65	gga-miR-458b-5	BICD2	BICD cargo adaptor 2
170	65	gga-miR-458b-5	NETO2	neuropilin and tolloid like 2
171	65	gga-miR-458b-5	GPT	glutamic--pyruvic transaminase
172	65	gga-miR-458b-5	CLOCK	clock circadian regulator
173	65	gga-miR-458b-5	NARF	nuclear prelamin A recognition factor
174	64	gga-miR-458b-5	RAD23B	RAD23 homolog B, nucleotide excision repair protein
175	64	gga-miR-458b-5	SSBP2	single stranded DNA binding protein 2
176	64	gga-miR-458b-5	EIF6	eukaryotic translation initiation factor 6
177	64	gga-miR-458b-5	SLC22A15	solute carrier family 22 member 15
178	64	gga-miR-458b-5	FGF10	fibroblast growth factor 10
179	64	gga-miR-458b-5	CUX2	cut like homeobox 2
180	63	gga-miR-458b-5	GPR107	G protein-coupled receptor 107
181	63	gga-miR-458b-5	IBTK	inhibitor of Bruton tyrosine kinase
182	63	gga-miR-458b-5	HS1BP3	HCLS1 binding protein 3
183	63	gga-miR-458b-5	BCL7A	BCL tumor suppressor 7A
184	63	gga-miR-458b-5	PPM1L	protein phosphatase, Mg2+/Mn2+ dependent 1L
185	63	gga-miR-458b-5	ZNF217L	zinc finger protein 217-like
186	63	gga-miR-458b-5	MTDH	metadherin
187	63	gga-miR-458b-5	MYO16	myosin XVI
188	63	gga-miR-458b-5	SDC1	syndecan 1
189	63	gga-miR-458b-5	LOC429054	protein FAM102B-like
190	63	gga-miR-458b-5	SHB	SH2 domain containing adaptor protein B

191	62	gga-miR-458b-5	EGLN3	egl-9 family hypoxia inducible factor 3
192	62	gga-miR-458b-5	TMEM70	transmembrane protein 70
193	62	gga-miR-458b-5	TCP11L2	t-complex 11 like 2
194	62	gga-miR-458b-5	RTKN2	rhotekin 2
195	62	gga-miR-458b-5	DLC1	DLC1 Rho GTPase activating protein
196	62	gga-miR-458b-5	VDAC2	voltage dependent anion channel 2
197	62	gga-miR-458b-5	B3GAT1L	beta-1,3-glucuronyltransferase 1-like
198	62	gga-miR-458b-5	SCN3B	sodium voltage-gated channel beta subunit 3
199	61	gga-miR-458b-5	TEAD1	TEA domain transcription factor 1
200	61	gga-miR-458b-5	SGPL1	sphingosine-1-phosphate lyase 1
201	61	gga-miR-458b-5	FXN	frataxin
202	61	gga-miR-458b-5	BEST1	bestrophin 1
203	61	gga-miR-458b-5	SALL3	spalt like transcription factor 3
204	61	gga-miR-458b-5	PLXNA1	plexin A1
205	61	gga-miR-458b-5	SLC33A1	solute carrier family 33 member 1
206	61	gga-miR-458b-5	CD99	CD99 molecule (Xg blood group)
207	60	gga-miR-458b-5	TMEM200A	transmembrane protein 200A
208	60	gga-miR-458b-5	NFAM1	NFAT activating protein with ITAM motif 1
209	60	gga-miR-458b-5	LHFPL6	LHFPL tetraspan subfamily member 6
210	60	gga-miR-458b-5	EP300	E1A binding protein p300
211	60	gga-miR-458b-5	PCNP	PEST proteolytic signal containing nuclear protein
212	60	gga-miR-458b-5	SHQ1	SHQ1, H/ACA ribonucleoprotein assembly factor
213	60	gga-miR-458b-5	SERTAD2	SERTA domain containing 2
214	60	gga-miR-458b-5	E4F1	E4F transcription factor 1
215	60	gga-miR-458b-5	ITPK1	inositol-tetrakisphosphate 1-kinase
216	60	gga-miR-458b-5	SBK2	SH3 domain binding kinase family member 2
217	60	gga-miR-458b-5	UNC5D	unc-5 netrin receptor D
218	60	gga-miR-458b-5	PTN	pleiotrophin
219	60	gga-miR-458b-5	CBR1	carbonyl reductase 1
220	59	gga-miR-458b-5	IRS4	insulin receptor substrate 4
221	59	gga-miR-458b-5	ATP23	ATP23 metallopeptidase and ATP synthase assembly facto
222	59	gga-miR-458b-5	LRIG1	leucine rich repeats and immunoglobulin like domains 1
223	59	gga-miR-458b-5	EVL	Enah/Vasp-like
224	59	gga-miR-458b-5	ZNF148	zinc finger protein 148
225	59	gga-miR-458b-5	KIAA0319	KIAA0319
226	59	gga-miR-458b-5	GJD4	gap junction protein delta 4
227	59	gga-miR-458b-5	MSL2	male-specific lethal 2 homolog (Drosophila)
228	58	gga-miR-458b-5	SOX5	SRY-box 5
229	58	gga-miR-458b-5	CLVS1	clavesin 1
230	58	gga-miR-458b-5	FOXK1	forkhead box K1
231	58	gga-miR-458b-5	LYN	LYN proto-oncogene, Src family tyrosine kinase
232	58	gga-miR-458b-5	KCNQ3	potassium voltage-gated channel subfamily Q member 3
233	58	gga-miR-458b-5	PITPNM3	PITPNM family member 3
234	58	gga-miR-458b-5	CNKSR2L	connector enhancer of kinase suppressor of Ras 2-like
235	58	gga-miR-458b-5	XRN1	5'-3' exoribonuclease 1
236	58	gga-miR-458b-5	CTGF	connective tissue growth factor
237	58	gga-miR-458b-5	PYGO1	pygopus family PHD finger 1
238	58	gga-miR-458b-5	SUMO1	small ubiquitin-like modifier 1

239	58	gga-miR-458b-5	TIMM23B	translocase of inner mitochondrial membrane 23 homolog
240	58	gga-miR-458b-5	VWA3A	von Willebrand factor A domain containing 3A
241	58	gga-miR-458b-5	LUC7L3	LUC7 like 3 pre-mRNA splicing factor
242	58	gga-miR-458b-5	PAFAH1B1	platelet activating factor acetylhydrolase 1b regulatory sub
243	58	gga-miR-458b-5	KCNJ8	potassium voltage-gated channel subfamily J member 8
244	57	gga-miR-458b-5	LMNB2	lamin B2
245	57	gga-miR-458b-5	PTCHD4	patched domain containing 4
246	57	gga-miR-458b-5	NIN	ninein
247	57	gga-miR-458b-5	MRVI1	murine retrovirus integration site 1 homolog
248	57	gga-miR-458b-5	ATP2B4	ATPase plasma membrane Ca2+ transporting 4
249	57	gga-miR-458b-5	SLC12A2	solute carrier family 12 member 2
250	57	gga-miR-458b-5	E2F3	E2F transcription factor 3
251	57	gga-miR-458b-5	HOMER1	homer scaffolding protein 1
252	57	gga-miR-458b-5	COA5	cytochrome c oxidase assembly factor 5
253	57	gga-miR-458b-5	GAB3	GRB2 associated binding protein 3
254	57	gga-miR-458b-5	VPS13A	vacuolar protein sorting 13 homolog A
255	57	gga-miR-458b-5	INPP5E	inositol polyphosphate-5-phosphatase E
256	57	gga-miR-458b-5	PPCS	phosphopantothenoylcysteine synthetase
257	56	gga-miR-458b-5	ANGPT2	angiopoietin 2
258	56	gga-miR-458b-5	MAP3K7	mitogen-activated protein kinase kinase kinase 7
259	56	gga-miR-458b-5	RPH3A	rabphilin 3A
260	56	gga-miR-458b-5	TMEM177	transmembrane protein 177
261	56	gga-miR-458b-5	PCSK7	proprotein convertase subtilisin/kexin type 7
262	56	gga-miR-458b-5	ADAMTS18	ADAM metallopeptidase with thrombospondin type 1 moti
263	56	gga-miR-458b-5	OFCC1	orofacial cleft 1 candidate 1
264	56	gga-miR-458b-5	ANKRD46	ankyrin repeat domain 46
265	56	gga-miR-458b-5	DKK2	dickkopf WNT signaling pathway inhibitor 2
266	56	gga-miR-458b-5	NPHP3	nephrocystin 3
267	55	gga-miR-458b-5	SGO2	shugoshin 2
268	55	gga-miR-458b-5	GMSRAL	granulocyte-macrophage colony-stimulating factor recepto
269	55	gga-miR-458b-5	MMP16	matrix metallopeptidase 16
270	55	gga-miR-458b-5	ADIPOQ	adiponectin, C1Q and collagen domain containing
271	55	gga-miR-458b-5	SLC8A1	solute carrier family 8 member A1
272	55	gga-miR-458b-5	BEGAIN	brain enriched guanylate kinase associated
273	55	gga-miR-458b-5	CCDC14	coiled-coil domain containing 14
274	55	gga-miR-458b-5	AGPAT2	1-acylglycerol-3-phosphate O-acyltransferase 2
275	55	gga-miR-458b-5	USP25	ubiquitin specific peptidase 25
276	55	gga-miR-458b-5	ELOVL4	ELOVL fatty acid elongase 4
277	55	gga-miR-458b-5	WWC2	WW and C2 domain containing 2
278	54	gga-miR-458b-5	DCLK1	doublecortin like kinase 1
279	54	gga-miR-458b-5	ZNF608	zinc finger protein 608
280	54	gga-miR-458b-5	WDR44	WD repeat domain 44
281	54	gga-miR-458b-5	CSGALNAC	chondroitin sulfate N-acetylgalactosaminyltransferase 2
282	54	gga-miR-458b-5	SAMD9L	sterile alpha motif domain containing 9 like
283	54	gga-miR-458b-5	SLC24A3	solute carrier family 24 member 3
284	53	gga-miR-458b-5	GPR141	G protein-coupled receptor 141
285	53	gga-miR-458b-5	TMEM33	transmembrane protein 33
286	53	gga-miR-458b-5	CRISP2	cysteine rich secretory protein 2

287	53	gga-miR-458b-5	HPS5	HPS5, biogenesis of lysosomal organelles complex 2 subu
288	53	gga-miR-458b-5	DENND3	DENN domain containing 3
289	53	gga-miR-458b-5	FRS2	fibroblast growth factor receptor substrate 2
290	53	gga-miR-458b-5	EDMTF4	epidermal differentiation protein starting with MTF motif
291	53	gga-miR-458b-5	ARHGAP18	Rho GTPase activating protein 18
292	53	gga-miR-458b-5	MANBA	mannosidase beta
293	53	gga-miR-458b-5	SEMA3C	semaphorin 3C
294	52	gga-miR-458b-5	KCNMA1	potassium calcium-activated channel subfamily M alpha 1
295	52	gga-miR-458b-5	C8H1orf123	chromosome 8 C1orf123 homolog
296	52	gga-miR-458b-5	COPE	coatomer protein complex subunit epsilon
297	52	gga-miR-458b-5	ANKRD13A	ankyrin repeat domain 13A
298	52	gga-miR-458b-5	SLC35E1	solute carrier family 35 member E1
299	52	gga-miR-458b-5	TXNDC5	thioredoxin domain containing 5
300	52	gga-miR-458b-5	SHC3	SHC adaptor protein 3
301	52	gga-miR-458b-5	PPHLN1	periphilin 1
302	52	gga-miR-458b-5	CNTN1	contactin 1
303	51	gga-miR-458b-5	FGB	fibrinogen beta chain
304	51	gga-miR-458b-5	LOC1008581	trichohyalin-like
305	51	gga-miR-458b-5	BYSL	bystin like
306	51	gga-miR-458b-5	DOK6	docking protein 6
307	51	gga-miR-458b-5	ZCCHC24	zinc finger CCHC-type containing 24
308	51	gga-miR-458b-5	IRAK2	interleukin 1 receptor associated kinase 2
309	51	gga-miR-458b-5	ZBTB5	zinc finger and BTB domain containing 5
310	50	gga-miR-458b-5	ATXN1	ataxin 1
311	50	gga-miR-458b-5	KLHL2	kelch like family member 2
312	50	gga-miR-458b-5	PCDH9	protocadherin 9
313	50	gga-miR-458b-5	FOXP3	forkhead box P3
314	50	gga-miR-458b-5	THL	tyrosine hydroxylase-like

Table S3 miR-458b-5p target genes predicted both by TargetScan and miRDB

Rank	Score	miRNA name	Gene symbo	Gene description
1	98	gga-miR-458b-5p	RASA2	RAS p21 protein activator 2
2	96	gga-miR-458b-5p	MON2	MON2 homolog, regulator of endosome-to-Golgi traffickin
3	96	gga-miR-458b-5p	STK24	serine/threonine kinase 24
4	95	gga-miR-458b-5p	CLIC4	chloride intracellular channel 4
5	94	gga-miR-458b-5p	CTNNB1	catenin beta 1
6	94	gga-miR-458b-5p	ZBTB10	zinc finger and BTB domain containing 10
7	94	gga-miR-458b-5p	ARFGEF1	ADP ribosylation factor guanine nucleotide exchange facto
8	94	gga-miR-458b-5p	BCL9	B-cell CLL/lymphoma 9
9	94	gga-miR-458b-5p	PHIP	pleckstrin homology domain interacting protein
10	93	gga-miR-458b-5p	RAB11FIP2	RAB11 family interacting protein 2
11	93	gga-miR-458b-5p	BICC1	BicC family RNA binding protein 1
12	92	gga-miR-458b-5p	BRD1	bromodomain containing 1
13	92	gga-miR-458b-5p	RNF111	ring finger protein 111
14	92	gga-miR-458b-5p	TXLNG	taxilin gamma
15	92	gga-miR-458b-5p	CACHD1	cache domain containing 1
16	92	gga-miR-458b-5p	ATP1B3	ATPase Na+/K+ transporting subunit beta 3
17	91	gga-miR-458b-5p	PHTF2	putative homeodomain transcription factor 2
18	90	gga-miR-458b-5p	SRSF3	serine and arginine rich splicing factor 3
19	90	gga-miR-458b-5p	FAM49B	family with sequence similarity 49 member B
20	90	gga-miR-458b-5p	PTPN12	protein tyrosine phosphatase, non-receptor type 12
21	89	gga-miR-458b-5p	CARF	calcium responsive transcription factor
22	89	gga-miR-458b-5p	DPYSL3	dihydropyrimidinase like 3
23	89	gga-miR-458b-5p	SUZ12	SUZ12, polycomb repressive complex 2 subunit
24	89	gga-miR-458b-5p	VPS54	VPS54, GARP complex subunit
25	88	gga-miR-458b-5p	GAB2	GRB2 associated binding protein 2
26	88	gga-miR-458b-5p	STRBP	spermatid perinuclear RNA binding protein
27	88	gga-miR-458b-5p	FOXO3	forkhead box O3
28	87	gga-miR-458b-5p	CDH2	cadherin 2
29	87	gga-miR-458b-5p	SNAP91	synaptosome associated protein 91
30	85	gga-miR-458b-5p	ELOVL6	ELOVL fatty acid elongase 6
31	85	gga-miR-458b-5p	ANKS1B	ankyrin repeat and sterile alpha motif domain containing 1I
32	84	gga-miR-458b-5p	ECT2	epithelial cell transforming 2
33	84	gga-miR-458b-5p	LHX9	LIM homeobox 9
34	84	gga-miR-458b-5p	GNA13	G protein subunit alpha 13
35	83	gga-miR-458b-5p	OTX2	orthodenticle homeobox 2
36	83	gga-miR-458b-5p	ZNF367	zinc finger protein 367
37	83	gga-miR-458b-5p	NF1	neurofibromin 1
38	82	gga-miR-458b-5p	GRM7	glutamate metabotropic receptor 7
39	82	gga-miR-458b-5p	FMR1	fragile X mental retardation 1
40	82	gga-miR-458b-5p	ANKIB1	ankyrin repeat and IBR domain containing 1
41	82	gga-miR-458b-5p	UBA6	ubiquitin like modifier activating enzyme 6
42	82	gga-miR-458b-5p	CNOT8	CCR4-NOT transcription complex subunit 8
43	81	gga-miR-458b-5p	PPM1E	protein phosphatase, Mg2+/Mn2+ dependent 1E
44	80	gga-miR-458b-5p	ARL8B	ADP ribosylation factor like GTPase 8B
45	79	gga-miR-458b-5p	HSPA4L	heat shock protein family A (Hsp70) member 4 like
46	79	gga-miR-458b-5p	BCORL1	BCL6 corepressor like 1

47	78	gga-miR-458b-5p	IPPK	inositol-pentakisphosphate 2-kinase
48	78	gga-miR-458b-5p	STK39	serine/threonine kinase 39
49	77	gga-miR-458b-5p	PPFIA1	PTPRF interacting protein alpha 1
50	77	gga-miR-458b-5p	SMARCA1	SWI/SNF related, matrix associated, actin dependent regul
51	76	gga-miR-458b-5p	BBX	BBX, HMG-box containing
52	76	gga-miR-458b-5p	TBL1XR1	transducin beta like 1 X-linked receptor 1
53	76	gga-miR-458b-5p	MYT1L	myelin transcription factor 1 like
54	76	gga-miR-458b-5p	LDLRAD4	low density lipoprotein receptor class A domain containing
55	75	gga-miR-458b-5p	PCDH10	protocadherin 10
56	75	gga-miR-458b-5p	ANKRD44	ankyrin repeat domain 44
57	75	gga-miR-458b-5p	BRD3	bromodomain containing 3
58	74	gga-miR-458b-5p	PTPRF	protein tyrosine phosphatase, receptor type F
59	74	gga-miR-458b-5p	LAMA5	laminin subunit alpha 5
60	73	gga-miR-458b-5p	DCBLD2	discoidin, CUB and LCCL domain containing 2
61	73	gga-miR-458b-5p	ARHGAP12	Rho GTPase activating protein 12
62	72	gga-miR-458b-5p	FCHO2	FCH domain only 2
63	72	gga-miR-458b-5p	HMGN5	high mobility group nucleosome binding domain 5
64	72	gga-miR-458b-5p	BRWD3	bromodomain and WD repeat domain containing 3
65	71	gga-miR-458b-5p	VCL	vinculin
66	71	gga-miR-458b-5p	PHF21B	PHD finger protein 21B
67	71	gga-miR-458b-5p	DR1	down-regulator of transcription 1
68	70	gga-miR-458b-5p	STT3B	STT3B, catalytic subunit of the oligosaccharyltransferase c
69	70	gga-miR-458b-5p	SOWAHC	sosondowah ankyrin repeat domain family member C
70	69	gga-miR-458b-5p	PLCXD3	phosphatidylinositol specific phospholipase C X domain co
71	69	gga-miR-458b-5p	PALLD	palladin, cytoskeletal associated protein
72	69	gga-miR-458b-5p	SREK1	splicing regulatory glutamic acid and lysine rich protein 1
73	69	gga-miR-458b-5p	ARL4A	ADP ribosylation factor like GTPase 4A
74	68	gga-miR-458b-5p	MET	MET proto-oncogene, receptor tyrosine kinase
75	68	gga-miR-458b-5p	TNPO1	transportin 1
76	68	gga-miR-458b-5p	FAM49A	family with sequence similarity 49 member A
77	68	gga-miR-458b-5p	POU2F1	POU class 2 homeobox 1
78	68	gga-miR-458b-5p	TSC1	TSC complex subunit 1
79	68	gga-miR-458b-5p	SNX13	sorting nexin 13
80	67	gga-miR-458b-5p	LIN28B	lin-28 homolog B
81	67	gga-miR-458b-5p	NOVA1	uncharacterized LOC423300
82	67	gga-miR-458b-5p	ALCAM	activated leukocyte cell adhesion molecule
83	66	gga-miR-458b-5p	SPRY3	sprouty RTK signaling antagonist 3
84	66	gga-miR-458b-5p	SMARCA2	SWI/SNF related, matrix associated, actin dependent regul
85	66	gga-miR-458b-5p	CUL4B	cullin 4B
86	66	gga-miR-458b-5p	BIRC6	baculoviral IAP repeat containing 6
87	65	gga-miR-458b-5p	PITPNC1	phosphatidylinositol transfer protein, cytoplasmic 1
88	65	gga-miR-458b-5p	KIF13A	kinesin family member 13A
89	65	gga-miR-458b-5p	BICD2	BICD cargo adaptor 2
90	65	gga-miR-458b-5p	NETO2	neuropilin and tolloid like 2
91	65	gga-miR-458b-5p	CLOCK	clock circadian regulator
92	64	gga-miR-458b-5p	FGF10	fibroblast growth factor 10
93	64	gga-miR-458b-5p	CUX2	cut like homeobox 2
94	63	gga-miR-458b-5p	IBTK	inhibitor of Bruton tyrosine kinase

95	63	gga-miR-458b-5p	BCL7A	BCL tumor suppressor 7A
96	63	gga-miR-458b-5p	MTDH	metadherin
97	62	gga-miR-458b-5p	DLC1	DLC1 Rho GTPase activating protein
98	61	gga-miR-458b-5p	TEAD1	TEA domain transcription factor 1
99	61	gga-miR-458b-5p	BEST1	bestrophin 1
100	61	gga-miR-458b-5p	SALL3	spalt like transcription factor 3
101	61	gga-miR-458b-5p	PLXNA1	plexin A1
102	60	gga-miR-458b-5p	TMEM200A	transmembrane protein 200A
103	60	gga-miR-458b-5p	PCNP	PEST proteolytic signal containing nuclear protein
104	60	gga-miR-458b-5p	SERTAD2	SERTA domain containing 2
105	60	gga-miR-458b-5p	UNC5D	unc-5 netrin receptor D
106	60	gga-miR-458b-5p	PTN	pleiotrophin
107	59	gga-miR-458b-5p	LRIG1	leucine rich repeats and immunoglobulin like domains 1
108	59	gga-miR-458b-5p	ZNF148	zinc finger protein 148
109	59	gga-miR-458b-5p	MSL2	male-specific lethal 2 homolog (Drosophila)
110	58	gga-miR-458b-5p	SOX5	SRY-box 5
111	58	gga-miR-458b-5p	KCNQ3	potassium voltage-gated channel subfamily Q member 3
112	58	gga-miR-458b-5p	CTGF	connective tissue growth factor
113	58	gga-miR-458b-5p	PYGO1	pygopus family PHD finger 1
114	58	gga-miR-458b-5p	LUC7L3	LUC7 like 3 pre-mRNA splicing factor
115	58	gga-miR-458b-5p	PAFAH1B1	platelet activating factor acetylhydrolase 1b regulatory sub
116	57	gga-miR-458b-5p	LMNB2	lamin B2
117	57	gga-miR-458b-5p	SLC12A2	solute carrier family 12 member 2
118	57	gga-miR-458b-5p	E2F3	E2F transcription factor 3
119	57	gga-miR-458b-5p	HOMER1	homer scaffolding protein 1
120	56	gga-miR-458b-5p	MAP3K7	mitogen-activated protein kinase kinase kinase 7
121	56	gga-miR-458b-5p	ADAMTS1	ADAM metallopeptidase with thrombospondin type 1 moti
122	56	gga-miR-458b-5p	ANKRD46	ankyrin repeat domain 46
123	55	gga-miR-458b-5p	MMP16	matrix metallopeptidase 16
124	55	gga-miR-458b-5p	SLC8A1	solute carrier family 8 member A1
125	55	gga-miR-458b-5p	USP25	ubiquitin specific peptidase 25
126	55	gga-miR-458b-5p	WWC2	WW and C2 domain containing 2
127	54	gga-miR-458b-5p	ZNF608	zinc finger protein 608
128	54	gga-miR-458b-5p	WDR44	WD repeat domain 44
129	54	gga-miR-458b-5p	SLC24A3	solute carrier family 24 member 3
130	53	gga-miR-458b-5p	TMEM33	transmembrane protein 33
131	53	gga-miR-458b-5p	SEMA3C	semaphorin 3C
132	52	gga-miR-458b-5p	SLC35E1	solute carrier family 35 member E1
133	52	gga-miR-458b-5p	TXNDC5	thioredoxin domain containing 5
134	52	gga-miR-458b-5p	CNTN1	contactin 1
135	51	gga-miR-458b-5p	FGB	fibrinogen beta chain
136	51	gga-miR-458b-5p	DOK6	docking protein 6
137	51	gga-miR-458b-5p	ZCCHC24	zinc finger CCHC-type containing 24
138	50	gga-miR-458b-5p	ATXN1	ataxin 1
139	50	gga-miR-458b-5p	KLHL2	kelch like family member 2

Note: The target score were predicted by miRDB.

Table S4 miRNAs targeting CTNNB1 predicted using TargetScan			
**miRNA**	**Position in the UTR**	**seed match**	**Gene symbol**
gga-miR-193a-3p	921-928	8mer	CTNNB1
gga-miR-193b-3p	921-928	8mer	CTNNB1
gga-miR-103-2-5p	15-21	7mer-m8	CTNNB1
gga-miR-107-5p	15-21	7mer-m8	CTNNB1
gga-miR-6596-3p	19-25	7mer-1A	CTNNB1
gga-miR-130b-5p	29-35	7mer-1A	CTNNB1
gga-miR-1717	31-37	7mer-1A	CTNNB1
gga-miR-1685-3p	43-49	7mer-m8	CTNNB1
gga-miR-6673-3p	52-58	7mer-1A	CTNNB1
gga-miR-6568-3p	60-66	7mer-m8	CTNNB1
gga-miR-1774	67-73	7mer-1A	CTNNB1
gga-miR-7455-3p	72-78	7mer-m8	CTNNB1
gga-miR-3525	77-83	7mer-m8	CTNNB1
gga-miR-6570-5p	88-94	7mer-m8	CTNNB1
gga-miR-1683	91-97	7mer-m8	CTNNB1
gga-miR-7458-3p	92-98	7mer-m8	CTNNB1
gga-miR-215-3p	100-106	7mer-1A	CTNNB1
gga-miR-6552-5p	101-107	7mer-1A	CTNNB1
gga-miR-16-1-3p	106-113	8mer	CTNNB1
gga-miR-16c-3p	106-113	8mer	CTNNB1
gga-miR-30a-3p	109-116	8mer	CTNNB1
gga-miR-6693-3p	109-116	8mer	CTNNB1
gga-miR-30e-3p	109-115	7mer-1A	CTNNB1
gga-miR-6701-3p	120-127	8mer	CTNNB1
gga-miR-757	127-133	7mer-m8	CTNNB1
gga-miR-1690-5p	129-135	7mer-1A	CTNNB1
gga-miR-7439-3p	131-137	7mer-m8	CTNNB1
gga-miR-6587-5p	131-137	7mer-1A	CTNNB1
gga-miR-6581-3p	133-139	7mer-1A	CTNNB1
gga-miR-206	151-157	7mer-m8	CTNNB1
gga-miR-1b-3p	151-157	7mer-m8	CTNNB1
gga-miR-1a-3p	151-157	7mer-m8	CTNNB1
gga-miR-6664-3p	152-159	8mer	CTNNB1
gga-miR-135a-2-3p	156-162	7mer-1A	CTNNB1
gga-miR-135a-3-3p	156-162	7mer-1A	CTNNB1
gga-miR-138-1-3p	179-185	7mer-m8	CTNNB1
gga-miR-6590-3p	189-195	7mer-1A	CTNNB1
gga-miR-144-3p	199-205	7mer-1A	CTNNB1
gga-miR-101-3p	199-205	7mer-1A	CTNNB1
gga-miR-6630-3p	210-216	7mer-m8	CTNNB1
gga-miR-6713-3p	222-228	7mer-m8	CTNNB1
gga-miR-6545-5p	225-231	7mer-1A	CTNNB1
gga-miR-2131-5p	225-231	7mer-m8	CTNNB1
gga-miR-7482-3p	226-232	7mer-m8	CTNNB1
gga-miR-6605-5p	226-232	7mer-m8	CTNNB1
gga-miR-302c-5p	238-245	8mer	CTNNB1

gga-miR-1685-3p	253-259	7mer-1A	CTNNB1
gga-miR-223	265-271	7mer-1A	CTNNB1
gga-miR-1781-5p	295-302	8mer	CTNNB1
gga-miR-7453-5p	304-310	7mer-1A	CTNNB1
gga-miR-1782	308-314	7mer-1A	CTNNB1
gga-miR-1467-5p	341-347	7mer-1A	CTNNB1
gga-miR-1452	368-374	7mer-m8	CTNNB1
gga-miR-6565-5p	368-374	7mer-m8	CTNNB1
gga-miR-1623	379-386	8mer	CTNNB1
gga-miR-6641-5p	397-403	7mer-1A	CTNNB1
gga-miR-1467-5p	398-404	7mer-m8	CTNNB1
gga-miR-1416-3p	417-424	8mer	CTNNB1
gga-miR-138-1-3p	422-428	7mer-1A	CTNNB1
gga-miR-6590-3p	423-429	7mer-1A	CTNNB1
gga-miR-7463-3p	424-430	7mer-1A	CTNNB1
gga-miR-1625-5p	440-446	7mer-m8	CTNNB1
gga-miR-1456-5p	443-449	7mer-m8	CTNNB1
gga-miR-126-5p	462-469	8mer	CTNNB1
gga-miR-7457-5p	503-509	7mer-1A	CTNNB1
gga-miR-1639	506-512	7mer-1A	CTNNB1
gga-miR-7469-5p	519-525	7mer-m8	CTNNB1
gga-miR-3531-3p	520-527	8mer	CTNNB1
gga-miR-1593	525-531	7mer-1A	CTNNB1
gga-miR-1555-3p	540-546	7mer-1A	CTNNB1
gga-miR-6552-3p	553-559	7mer-1A	CTNNB1
gga-miR-302c-5p	555-561	7mer-1A	CTNNB1
gga-miR-7473-5p	580-586	7mer-m8	CTNNB1
gga-miR-458b-5p	590-596	7mer-m8	CTNNB1
gga-miR-21-3p	641-648	8mer	CTNNB1
gga-miR-6571-3p	664-670	7mer-m8	CTNNB1
gga-miR-429-3p	673-679	7mer-1A	CTNNB1
gga-miR-200b-3p	673-679	7mer-1A	CTNNB1
gga-miR-124c-5p	687-693	7mer-1A	CTNNB1
gga-miR-1796	700-706	7mer-m8	CTNNB1
gga-miR-1416-5p	715-721	7mer-1A	CTNNB1
gga-miR-1807	766-772	7mer-1A	CTNNB1
gga-miR-6544-5p	767-773	7mer-1A	CTNNB1
gga-miR-1559-3p	779-785	7mer-1A	CTNNB1
gga-let-7k-3p	799-806	8mer	CTNNB1
gga-let-7f-3p	799-806	8mer	CTNNB1
gga-let-7a-3p	799-806	8mer	CTNNB1
gga-let-7j-3p	800-806	7mer-1A	CTNNB1
gga-miR-1463	816-822	7mer-1A	CTNNB1
gga-miR-1625-5p	823-829	7mer-1A	CTNNB1
gga-miR-1612	835-841	7mer-m8	CTNNB1
gga-miR-6701-3p	854-860	7mer-m8	CTNNB1
gga-miR-1306-5p	863-869	7mer-1A	CTNNB1
gga-miR-1667-3p	876-882	7mer-m8	CTNNB1

gga-miR-200b-5p	924-931	8mer	CTNNB1
gga-miR-133c-5p	963-969	7mer-m8	CTNNB1
gga-miR-7459-3p	978-984	7mer-1A	CTNNB1
gga-miR-6700-5p	979-985	7mer-1A	CTNNB1
gga-miR-6547-5p	979-985	7mer-1A	CTNNB1
gga-miR-187-5p	991-997	7mer-m8	CTNNB1
gga-miR-1556	995-1001	7mer-m8	CTNNB1
gga-miR-6566-3p	996-1002	7mer-m8	CTNNB1
gga-miR-214	997-1003	7mer-m8	CTNNB1
gga-miR-383-3p	999-1006	8mer	CTNNB1
gga-miR-3594-5p	1000-1006	7mer-1A	CTNNB1
gga-miR-1697	1018-1025	8mer	CTNNB1
gga-miR-153-5p	1024-1030	7mer-m8	CTNNB1
gga-miR-460a-5p	1027-1033	7mer-m8	CTNNB1
gga-miR-217-5p	1028-1034	7mer-m8	CTNNB1
gga-miR-1796	1030-1036	7mer-1A	CTNNB1
gga-miR-1620	1051-1057	7mer-m8	CTNNB1
gga-miR-7463-3p	1054-1061	8mer	CTNNB1
gga-miR-1643-3p	1061-1067	7mer-1A	CTNNB1

Table S5 miRNAs targeting CTNNB1 predicted using miRDB

Target rank	Target score	miRNA name	Gene symbol	Gene description
1	97	gga-miR-9b-3p	CTNNB1	catenin beta 1
2	96	gga-miR-1625-5p	CTNNB1	catenin beta 1
3	94	gga-miR-7463-3p	CTNNB1	catenin beta 1
4	94	gga-miR-458b-5p	CTNNB1	catenin beta 1
5	91	gga-let-7f-3p	CTNNB1	catenin beta 1
6	91	gga-let-7a-3p	CTNNB1	catenin beta 1
7	91	gga-let-7k-3p	CTNNB1	catenin beta 1
8	89	gga-miR-1556	CTNNB1	catenin beta 1
9	88	gga-miR-1623	CTNNB1	catenin beta 1
10	83	gga-miR-200b-5p	CTNNB1	catenin beta 1
11	82	gga-miR-1467-5p	CTNNB1	catenin beta 1
12	78	gga-miR-1667-3p	CTNNB1	catenin beta 1
13	77	gga-miR-1559-3p	CTNNB1	catenin beta 1
14	77	gga-miR-214	CTNNB1	catenin beta 1
15	75	gga-miR-133c-5p	CTNNB1	catenin beta 1
16	72	gga-miR-6547-5p	CTNNB1	catenin beta 1
17	72	gga-miR-1416-3p	CTNNB1	catenin beta 1
18	72	gga-miR-6700-5p	CTNNB1	catenin beta 1
19	70	gga-miR-12280-5p	CTNNB1	catenin beta 1
20	68	gga-miR-12209-5p	CTNNB1	catenin beta 1
21	68	gga-miR-383-3p	CTNNB1	catenin beta 1
22	66	gga-miR-193a-3p	CTNNB1	catenin beta 1
23	66	gga-miR-193b-3p	CTNNB1	catenin beta 1
24	66	gga-miR-12278-3p	CTNNB1	catenin beta 1
25	64	gga-miR-6566-3p	CTNNB1	catenin beta 1
26	62	gga-miR-1697	CTNNB1	catenin beta 1
27	62	gga-miR-1643-3p	CTNNB1	catenin beta 1
28	60	gga-let-7j-3p	CTNNB1	catenin beta 1
29	60	gga-miR-6590-3p	CTNNB1	catenin beta 1
30	59	gga-miR-1456-5p	CTNNB1	catenin beta 1
31	59	gga-miR-122b-5p	CTNNB1	catenin beta 1
32	58	gga-miR-460a-5p	CTNNB1	catenin beta 1
33	55	gga-miR-7459-3p	CTNNB1	catenin beta 1
34	55	gga-miR-124c-5p	CTNNB1	catenin beta 1
35	55	gga-miR-21-3p	CTNNB1	catenin beta 1
36	53	gga-miR-138-1-3p	CTNNB1	catenin beta 1
37	53	gga-miR-12286-3p	CTNNB1	catenin beta 1
38	53	gga-miR-3531-3p	CTNNB1	catenin beta 1
39	52	gga-miR-7457-5p	CTNNB1	catenin beta 1

Table S6 miRNAs targeting CTNNB1 predicted both by TargetScan and miRDB

Target rank	Target score	miRNA name	Gene symbol	Gene description
1	96	gga-miR-1625-5p	CTNNB1	catenin beta 1
2	94	gga-miR-458b-5p	CTNNB1	catenin beta 1
3	94	gga-miR-7463-3p	CTNNB1	catenin beta 1
4	91	gga-let-7f-3p	CTNNB1	catenin beta 1
5	91	gga-let-7a-3p	CTNNB1	catenin beta 1
6	91	gga-let-7k-3p	CTNNB1	catenin beta 1
7	89	gga-miR-1556	CTNNB1	catenin beta 1
8	88	gga-miR-1623	CTNNB1	catenin beta 1
9	83	gga-miR-200b-5p	CTNNB1	catenin beta 1
10	82	gga-miR-1467-5p	CTNNB1	catenin beta 1
11	78	gga-miR-1667-3p	CTNNB1	catenin beta 1
12	77	gga-miR-1559-3p	CTNNB1	catenin beta 1
13	77	gga-miR-214	CTNNB1	catenin beta 1
14	75	gga-miR-133c-5p	CTNNB1	catenin beta 1
15	72	gga-miR-6547-5p	CTNNB1	catenin beta 1
16	72	gga-miR-1416-3p	CTNNB1	catenin beta 1
17	72	gga-miR-6700-5p	CTNNB1	catenin beta 1
18	68	gga-miR-383-3p	CTNNB1	catenin beta 1
19	66	gga-miR-193a-3p	CTNNB1	catenin beta 1
20	66	gga-miR-193b-3p	CTNNB1	catenin beta 1
21	64	gga-miR-6566-3p	CTNNB1	catenin beta 1
22	62	gga-miR-1697	CTNNB1	catenin beta 1
23	62	gga-miR-1643-3p	CTNNB1	catenin beta 1
24	60	gga-let-7j-3p	CTNNB1	catenin beta 1
25	60	gga-miR-6590-3p	CTNNB1	catenin beta 1
26	59	gga-miR-1456-5p	CTNNB1	catenin beta 1
27	58	gga-miR-460a-5p	CTNNB1	catenin beta 1
28	55	gga-miR-7459-3p	CTNNB1	catenin beta 1
29	55	gga-miR-124c-5p	CTNNB1	catenin beta 1
30	55	gga-miR-21-3p	CTNNB1	catenin beta 1
31	53	gga-miR-138-1-3p	CTNNB1	catenin beta 1
32	53	gga-miR-3531-3p	CTNNB1	catenin beta 1
33	52	gga-miR-7457-5p	CTNNB1	catenin beta 1

Note: The target score were predicted by miRDB.
